# Single-Cell Transcriptomics on *PRPF31*-Mutated Retinal Organoids Reveal Early Müller Glial Activation and Progressive Photoreceptor Degeneration

**DOI:** 10.3390/biomedicines14010045

**Published:** 2025-12-24

**Authors:** Alessandro Bellapianta, Jingjing Qi, Michele Giugliano, Sara Ouaidat, Rana El Rawas, Matthias Bolz, Ahmad Salti

**Affiliations:** 1 Research Group Cellular and Molecular Ophthalmology, University Clinic for Ophthalmology and Optometry, Johannes Kepler University Linz, Kepler University Hospital, Altenberger Strasse 69, 4040 Linz and Krankenhausstrasse 5, 4020 Linz, Austria; 2 Tumor Epigenetics Laboratory, Johannes Kepler University Linz, Altenbergerstraße 69, 4040 Linz, Austria; 3 Department of Biomedical, Metabolic and Neural Sciences, University of Modena and Reggio Emilia, I-41125 Modena, Italy; 4 Division of Psychiatry I, Department of Psychiatry, Psychotherapy, Psychosomatics and Medical Psychology, Medical University Innsbruck, 6020 Innsbruck, Austria

**Keywords:** *PRPF31*, retinitis pigmentosa, retinal dystrophies, retinal organoids, induced pluripotent stem cells, single-cell RNA sequencing, Müller glia, photoreceptor degeneration, retinal ganglion cells

## Abstract

**Background**: Retinitis pigmentosa (RP) encompasses a group of inherited retinal disorders characterized by progressive degeneration of rod and cone photoreceptors, leading to vision loss. Among RP subtypes, RP11 is linked to mutations in *PRPF31*, a key spliceosome component, resulting in retinal cell dysfunction. Although *PRPF31* is ubiquitously expressed, its mutations predominantly impact retinal cells, leading to the progressive loss of photoreceptors. Despite significant progress, studies have focused on photoreceptor and retinal pigment epithelium dysfunction in late disease stages, leaving early molecular events and the involvement of other retinal cell types unresolved. Moreover, comprehensive single-cell analyses capturing dynamic transcriptional changes across all retinal populations at early and late differentiation stages are still lacking. **Methods:** Using patient-derived retinal organoids (ROs), this study investigates the impact of *PRPF31*-RP11 mutation through a series of morphological, functional, molecular, and transcriptomics analyses. **Results:**. Single-cell RNA sequencing revealed dynamic gene expression related to early Müller glia activation, retinal ganglion cell distress, and progressive photoreceptor degeneration. Findings identify dysregulated molecular pathways associated with phototransduction, oxidative stress, and inflammation. **Conclusions:** Our results support a specific RO model of RP11 in which *PRPF31* mutation recapitulate in vitro key features of RP, while simultaneously eliciting compensatory or modulatory responses in other retinal cell types.

## 1. Introduction

Retinitis pigmentosa (RP) is a group of inherited retinal disorders characterized by the progressive loss of rod photoreceptors, followed by secondary cone photoreceptor degeneration. RP affects ~1:4000 people worldwide [1], and patients experience a triphasic symptomatic course, with night blindness and peripheral visual field loss in the early stages, followed by loss of central vision and color vision in later stages [2,3,4]. These conditions display diverse genetic causes and clinical manifestations, with varying ages of onset, disease progression, and patterns of visual impairment [5]. Non-syndromic RP has been linked to mutations in over 85 causative genes/loci, and follows an autosomal recessive (arRP, 15–25% of cases), autosomal dominant (adRP 5–20% of cases), or X-linked (xlRP, 5–15% of cases) mode of inheritance [6,7]. Recent next-generation sequencing studies have substantially improved the diagnostic yield in RP, with most cohorts now reporting ~60–75% solved cases [8]. A minority of cases remain unsolved, often due to deep-intronic or structural variants not captured by conventional panels [9].

Among the numerous genes associated with RP, mutations in the *PRPF31* gene are responsible for RP type 11, an autosomal dominant form of the disease [10]. *PRPF31* encodes a critical component of the spliceosome, a macromolecular complex responsible for pre-mRNA splicing, suggesting that dysregulation of pre-mRNA splicing may be a key pathogenic mechanism in RP11 [11]. Although *PRPF31* is ubiquitously expressed, its mutations predominantly impact retinal cells, leading to the progressive loss of photoreceptors [12]. This tissue-specific vulnerability remains a central question in understanding the molecular pathogenesis of RP11, as studies indicate that the haploinsufficiency of *PRPF31*—where one functional allele is not sufficient to maintain normal cellular processes—leads to a reduction in functional protein, which disrupts the splicing of numerous genes crucial for retinal homeostasis [13]. As a result, dysfunction of the retinal pigment epithelium (RPE) impairs its ability to support photoreceptors, ultimately causing degeneration of the overlying retinal cells [14].

The advent of human-induced pluripotent stem cell (hiPSC) technology has provided unprecedented opportunities to model RP and other retinal diseases using patient-specific cells [3,15]. hiPSC-derived retinal organoids (ROs) are three-dimensional structures that recapitulate many of the key features of human retinal development. These organoids reproduce the layered organization of the neural retina and develop an adjacent retinal pigment epithelium (RPE) layer, enabling the modeling of disease progression in a physiologically relevant context [16]. In particular, their utility in studying neuroretinal disorders and evaluating retinal prosthetic interfaces further highlights their translational promise [17,18,19]. In RP11, ROs derived from patients’ iPSCs have been used to study the impact of *PRPF31* mutations on retinal development and function. For instance, *PRPF31*-deficient organoids exhibit altered cellular morphology, including defects in cilia, abnormal nuclear morphology, and increased apoptosis, which mirror the progressive degeneration seen in RP11 patients [10]. The scalability of organoids and their ability to mimic human retinogenesis make them an ideal tool for identifying novel therapeutic targets, testing potential treatments, and understanding disease pathophysiology in patient-specific contexts [16].

Despite significant progress, key gaps remain in our understanding of *PRPF31*-associated retinitis pigmentosa. Previous studies have primarily focused on photoreceptor and RPE dysfunction at late disease stages, leaving early molecular events and the involvement of other retinal cell types poorly defined [13,20,21]. The retina’s tissue-specific vulnerability to *PRPF31* mutations, despite its ubiquitous expression, remains incompletely explained, and the temporal sequence linking early cellular distress to eventual photoreceptor loss is largely unresolved. Moreover, while hiPSC-derived ROs have successfully modeled structural defects and apoptosis, comprehensive single-cell analyses capturing dynamic transcriptional changes across all retinal populations at early and late differentiation stages have been lacking. Thus, the aim of this study is to systematically dissect the degenerative trajectory of *PRPF31* mutation, using patient-derived ROs as human in vitro models. For this purpose, we generated ROs from commercially available hiPSC line derived from a patient carrying a *PRPF31*-RP11 mutation, as well as a healthy control line. These ROs were analyzed at both early and late stages of differentiation in order to capture dynamic changes and progressive degenerative and functional processes linked to the *PRPF31* mutation. In addition, we applied single-cell RNA sequencing (scRNA-seq) to understand at the single-cell level the discrete cellular and molecular changes across early and late differentiation stages, as well as across the various retinal cell populations. Our results on the investigated patient line suggested an early-stage Müller glia activation accompanied by transcriptional distress in retinal ganglion cells (RGCs), leading progressively to severe rod–cone photoreceptor degeneration. Moreover, our integrative approach identified, at least in this patient line, cell-type-specific molecular pathways significantly dysregulated by the *PRPF31* mutation, notably those related to phototransduction, oxidative stress, and inflammatory signaling.

## 2. Materials and Methods

### 2.1. Cell Lines and Cell Culture

hiPSCs derived from a very severe RP patient carrying a *PRPF31* mutation (19q13.42; *PRPF31*: c.1115_1125 del11; UNEWi004-A; EBiSC), as well as a hiPSC line derived from an unaffected subject (WTSIi004-A; EBiSC), were cultured under feeder-free conditions in StemMACs iPS-Brew XF medium (130-104-360; Miltenyi Biotec, Bergisch Gladbach, Germany) supplemented with 1% Penicillin/Streptomycin (P/S) (15070063; Thermo Fisher Scientific, Dreieich, Germany) on Matrigel-coated plates (354277; Corning, Kaiserslautern, Germany). The iPSCs were maintained in a humidified incubator at 37 °C with 5% CO_2_, and the medium was changed daily. Cells were passaged every 4–5 days when they reached approximately 80% confluency. For passaging, cells were dissociated using Accutase (A1110501, Thermo Fisher Scientific, Dreieich, Germany) for 5 min at 37 °C. The dissociated cells were then replated at a 1:6 to 1:10 ratio on fresh Matrigel-coated plates in StemMACs iPS-Brew XF medium supplemented with 10 µM Y-27632 (ROCK inhibitor, 1254; Tocris Bioscience, Birstol, UK) to enhance cell survival after passaging. Routine monitoring of the iPSCs was performed to ensure that the cells maintained a stable, undifferentiated state. Mycoplasma contamination was tested monthly using the Mycoplasma Detection Kit (G238; abm, Richmond, BC, Canada), and only mycoplasma-free cell cultures were used in downstream applications. Cryopreservation was performed using 2× Freezing media diluted 1:2 with the culture medium (80% Knock-Out Serum Replacement (KOSR) (10829220; Gibco^®^, Thermo Fisher Scientific, Dreieich, Germany) and 20% DMSO (A36720050; ITW Reagents, Monza, Italy) at a density of 1 × 10^6^ cells per cryovial. For thawing, vials were rapidly thawed in a 37 °C water bath and immediately plated onto Matrigel (354277, Corning, Kaiserslautern, Germany)-coated plates in iPS-Brew medium containing 10 µM Y-27632 to promote survival.

### 2.2. Retinal Organoid Differentiation

ROs were generated from iPSCs following published methods [22,23] with modifications. iPSC colonies were grown to 70–80% confluency before initiation of the differentiation process. Upon reaching the desired confluency, the colonies were treated with Dispase (2 mg/mL; 354235, Corning, Kaiserslautern, Germany). Once the edges of the colonies curled slightly, they were washed in 1 × PBS (PBS-1A; Capricorn Scientific, Ebsdorfergrund, Germany) and transferred to a T75 flask (10364131; Thermo Fisher Scientific, Dreieich, Germany) containing a 3:1 mixture of iPSBrew and Neural Induction Medium (NIM) (95.9% DMEM (12430054; Gibco^®^, Thermo Fisher Scientific, Dreieich, Germany); 1% N-2 (17502048; Gibco^®^, Thermo Fisher Scientific, Dreieich, Germany); 1% MEM-NEAA (11140050; Gibco^®^ Thermo Fisher Scientific, Dreieich, Germany; 1% GlutaMax (35050-061; Gibco^®^, Thermo Fisher Scientific, Dreieich, Germany); 1% P/S; 0.1% Heparin (2 μg/mL) (07980; Stemcell Technologies) to initiate neural differentiation. For the first six days, the medium was gradually switched from iPSBrew to NIM, facilitating the transition from a pluripotent state to neural induction. On day 6, the media was switched to fresh NIM supplemented with 1.5 nM bone morphogenetic protein 4 (BMP4) (GF302; Sigma-Aldrich^®^, Merck KGaA, Darmstadt, Germany) to promote the formation of neural precursors and optic vesicle-like structures. By day 7, formed embryoid bodies (EBs) were plated onto GFR-Matrigel-coated (Corning, Kaiserslautern, Germany; 354230) culture dishes to encourage the development of retinal progenitors. Half of the medium was refreshed with NIM every other day to wean the concentration of BMP4 until day 16, when it was switched to Retinal Differentiation Medium (RDM) (71% DMEM; 24% DMEM/F12 (11330057; Gibco^®^, Thermo Fisher Scientific, Dreieich, Germany); 2% B27™ Supplement minus Vitamin A (11500446; Gibco^®^, Thermo Fisher Scientific, Dreieich, Germany); 1% MEM NEAA; 1% GlutaMAX; 1% P/S), which supports the specification of retinal cells. On day 30, optic vesicle-like structures observed under a brightfield microscope (AE2000, Motic, Wetzlar, Germany) were manually collected using 23G Micro scissors (556008; HasaOptix, Brussels, Belgium) and resuspended in RDM and transferred to 48-well plates (CC7682-7548, Starlab, Hamburg, Germany) coated with Anti-Adherence Rinsing Solution (07010, Stemcell Technologies, Köln, Germany), allowing them to remain in suspension and continue their differentiation into 3D retinal organoids. Cultures were maintained on an orbital shaker set at low speed (~40 rpm) to ensure gentle agitation and prevent organoid attachment. The organoids were maintained in RDM until day 41, the point at which the medium was changed to Retinal Culture Medium 2 (RC2 medium) (57.2% DMEM; 26% DMEM/F12; 10% FBS; 2% B27™ Supplement; 1% P/S; 1% MEM-NEAA; 1% GlutaMax; 0.2% Taurine (50 mM) (T0625; Sigma-Aldrich^®^, Merck KGaA, Darmstadt, Germany), which supports further retinal lamination and photoreceptor maturation. Retinoic acid 1 μM (R2625; Signa-Aldrich^®^, Merck KGaA, Darmstadt, Germany) was added to the medium starting on day 61 to enhance photoreceptor differentiation, particularly rod and cone photoreceptors. On day 91, the medium was switched to Retinal Culture Medium 1 RC1 (85.8% DMEM/F12; 10% FBS (10439001; Gibco^®^, Thermo Fisher Scientific, Dreieich, Germany); 1% N-2; 1% MEM-NEAA; 1% GlutaMAX; 1% P/S; 0.2% Taurine (50 mM), which supports the final stages of organoid maturation. The organoids were maintained in this medium until day 250. Retinal organoids were collected and further analyzed at various time points.

### 2.3. Cells or Organoid Fixation and Cryoprotection

Cells or organoids were incubated in 4% paraformaldehyde (PFA) (158127; Sigma-Aldrich^®^, Merck KGaA, Darmstadt, Germany) for 15 min at room temperature. Following fixation, cells or organoids were washed multiple times with 1 × phosphate-buffered saline (PBS) (PBS-1A; Capricorn Scientific, Ebsdorfergrund, Germany) to remove any residual fixative. For cryoprotection, the organoids were incubated overnight in 30% sucrose (6104-; Sigma-Aldrich^®^, Merck KGaA, Darmstadt, Germany) in 1 × PBS at 4° C. Then, the organoids were embedded in 7.5% gelatin (4274.1; Roth) in 10% sucrose to provide support during sectioning. The gelatin-embedded organoids were flash-frozen in isopentane cooled at −55/−65 °C with dry ice and stored at −80 °C until sectioning. Organoids were sliced into thin sections (10–20 µm) using a Cryotome (Leica 3050S, Wetzlar, Germania) set to a temperature of approximately −20 °C. The sections were collected onto Superfrost Plus slides (10149870; FisherScientific, Schwerte, Germany) and stored at −20 °C for further analysis.

### 2.4. Immunostaining, Imaging, and Cell Counting

Organoid sections or coverslips were permeabilized with 0.3% Triton-X-100 (93443; Signa-Aldrich) in TBS (J60764.K2; Thermo Fisher Scientific, Dreieich, Germany) 1× for 15 min at RT. The sections were then incubated in blocking buffer (10% goat serum (BYT-ORB867942-100; BIOZOL Diagnostica, Hambrug, Germany) in 1 × TBS+ 0.025% Triton-X-100) for 1 h at RT. After blocking, the sections were incubated overnight in a humidified chamber at 4 °C with primary antibodies specific to the proteins of interest (Supplementary Table S1). Primary antibodies were diluted in incubation solution (10% goat serum in 1 × TBS + 0.025% Triton-X-100). The following day, the sections were washed three times with 1 × TBS and incubated with secondary antibodies (Supplementary Table S2), diluted 1:500 in incubation solution, for 1 h at RT in the dark. After further washing steps with TBST, DAPI (4′,6-diamidino-2-phenylindole) (H3570; Life Technologies, Darmstadt, Germany) (1:2000) was added to the sections and incubated for 5 min to stain the nuclei. After washing, the sections were mounted on glass slides (0111560; Marienfeld Superior, Lauda-Königshofen, Germany) using AquaPoli Mount (Clini Sciences, Biotrend Chemikalien, Köln, Germany; 18606-20). Imaging was performed using a Leica Stellaris 5 confocal microscope and a Leica Thunder wide-field fluorescence microscope with computational clearing and deconvolution using the LAS X software (version 5.3.1, Leica Microsystems, Wetzlar, Germany). The images were processed using Fiji/ImageJ software (2.9.0) for analysis of protein localization and expression and Huygens Professional (16.10) for deconvolution. Quantification of the number of total cells (DAPI) and cells immunoreactive for specific markers was performed by manual counting using the Fiji/ImageJ software (2.9.0). A minimum of three acquisitions from three independent differentiation experiments were counted for each condition.

### 2.5. Flow Cytometry

Flow cytometry analysis of stem cell surface markers was performed using anti-human TRA-1-60-PE antibody (330609; BioLegend, Koblenz, Germany). Human iPSCs were harvested using Accutase (A1110501, Thermo Fisher Scientific, Dreieich, Germany). For staining, 1 × 10^5^ to 1 × 10^6^ cells (determined using the Countess II FL (Invitrogen^®^, Thermo Fisher Scientific, Dreieich, Germany)) per sample were resuspended in FACS buffer (PBS, pH 7.2, containing 0.5% BSA (B14; Thermo Fisher Scientific, Dreieich, Germany) and 2 mM EDTA (17892; Thermo Fisher Scientific, Dreieich, Germany). Subsequently, TRA-1-60-PE antibody (1:10 dilution) was added and incubated for 10 min at 4 °C in the dark. Post-incubation, cells were washed and resuspended in FACS buffer. Samples were kept on ice and immediately processed for flow cytometry analysis. Flow cytometry data were acquired using the CytoFlex flow cytometer (Beckman Coulter, Inc., Brea, CA, USA), capturing 10,000 events per sample. Data processing and analysis were conducted using CytExpert software version 2.6 (Tree Star, Inc., Ashland, OR, USA) and Kaluza software V 2.1 (Beckman Coulter, Inc., Brea, CA, USA).

### 2.6. RNA Extraction and cDNA Synthesis

Organoids at different time points were dissociated and homogenized using TRIzol reagent (1596026; Thermo Fisher Scientific, Dreieich, Germany), and RNA extraction was performed following the manufacturer’s protocol. RNA cleanup procedure was performed using the Monarch^®^ RNA Cleanup Kit Protocol (T2030L; New England Biolabs, Frankfurt am Main, Germany), and the RNA was eluted in 20 µL of RNase-free water (AM9932; LifeTech, Düsseldorf, Germany). The final RNA concentration and purity were assessed using a Nanodrop spectrophotometer (Nanodrop One C; Thermo Fisher Scientific, Dreieich, Germany), measuring absorbance at 260 nm and 280 nm. Then, 1000 ng of the isolated RNA was further used for cDNA synthesis using the LunaScript RT SuperMix Kit (E3010; New England Biolabs, Frankfurt am Main, Germany) according to the manufacturer’s instructions. The synthesized cDNA was stored at −20 °C for downstream applications.

### 2.7. RTqPCR

Gene expression analysis was carried out using the Luna Universal qPCR Master Mix (M3003; New England Biolabs^®^) on a CFX Opus 96 Real-Time PCR System (12011319; Bio-Rad). A list of the selected primers is provided in Supplementary Table S3. Primers were purchased from Sigma-Aldrich^®^/Merck, KGaA, Darmstadt, Germany. The RTqPCR run was set with an initiation temperature of 95 °C, followed by 40 cycles of denaturation (95 °C; 15 s) and extension (60 °C, 30 s + plate read), and ending with the melting curve (60–95 °C). Fold changes were calculated following the ΔΔCt method and using GAPDH as a reference gene.

### 2.8. PCR Amplification of PRPF31 Gene

Genomic DNA was extracted from hiPSCs and used as a template to verify the presence of the *PRPF31* c.1115_1125del11 mutation. Specific forward (TGGATGGACAGCGGAAGAAG) and reverse (CTCGTTTACCTGTGTCTGCC) primers were designed to flank the mutation site. PCR amplification was carried out in a 25 µL reaction using a high-fidelity DNA polymerase (M0491S; New England Biolabs, Frankfurt am Main, Germany) under standard cycling conditions (initial denaturation at 95 °C, followed by 35 cycles of 95 °C denaturation, 60 °C annealing, and 72 °C extension). The PCR products were separated on a 2% agarose gel (LQ1630440500; LabConsulting, Vienna, Austria) and visualized under UV light after ethidium bromide (15585011; Thermo Fisher Scientific, Dreieich, Germany) staining.

### 2.9. Multi-Electrode Array (MEA) Recording

To evaluate spontaneous extracellular electrical activity in retinal organoids (ROs), recordings were conducted at day 60 of differentiation using a multi-electrode array (MEA) system. A threshold of 4.5× the SD of the baseline noise was applied for spike detection. Recordings were conducted in 10 independent RO trials per condition, and spontaneous activity was consistently detected in three organoids per group, which were included in the analysis. Signal amplitude in ROs was generally low, limiting the number of analyzable recordings. Prior to recording, MEA chips (60MEA200/30iR-ITO, Multichannel Systems, Harvard Instruments, Reutlingen, Germany) were coated with 200 µg/mL growth factor-reduced (GFR) Matrigel (354230; Corning, Kaiserslautern, Germany) and incubated at 37 °C for 1 h, followed by a 30 min adaptation period at room temperature. A radial incision with a surgical blade was performed to expose the inner retinal layers, particularly the RGCs-rich regions. The dissected organoids were then carefully positioned onto the MEA chips, ensuring optimal contact between the RGC layer and the electrode array. To promote stable attachment, organoids were maintained on the MEA for at least 24 h in a humidified incubator set at 37 °C with 5% CO_2_ prior to electrophysiological recording during the following days. Recordings were performed using the MEA2100-Mini-System (Multichannel Systems, Harvard Instruments, Reutlingen, Germany) in conjunction with the MCS Experimenter software (version 2.21.1). A band-pass filter with cutoff frequencies of 0.1 Hz and 5 kHz was applied to isolate relevant neuronal signals. Each recording session lasted 5 min, during which spontaneous electrical activity was captured. To maintain optimal culture conditions and nutrient availability, the culture medium was refreshed every 15 min throughout the recording sessions. All electrophysiological recordings were conducted within a controlled environment, maintaining a constant temperature of 37 °C and 5% CO_2_ to preserve organoid viability and functionality during data acquisition.

### 2.10. Single-Cell RNA Sequencing (scRNAseq)

Six ROs at culture day (D)85 and 285, from three parallel and independent differentiation experiments, were pooled. Organoids were washed in DPBS (no calcium, no magnesium) (14190144; Thermo Fisher Scientific, Dreieich, Germany). For both time conditions, Trypsin (P10-0235SP; PAN Biotech, Aidenbach, Germany) was added, then incubated at 37 °C on a nutator for 7.5 min. The organoids were then dissociated to single cells by pipetting up and down (4/5 times). The Trypsin was deactivated by the addition of double the amount of DMEM (12430054; Gibco^®^, Thermo Fisher Scientific, Dreieich, Germany), and the cell suspension was filtered through a 40 µm cell strainer (15342931, Thermo Fisher Scientific, Dreieich, Germany). The dissociation was complete within 20 min after Trypsin addition. The number of cell survival was determined by a viability assessment with Trypan Blue (T10282; Fischer Scientific) in a Countess II FL (Countess II FL; Invitrogen^®^, Thermo Fisher Scientific, Dreieich, Germany). Only samples with more than 90% viability were selected for the analysis. Finally, cells were spun down at 700 g for 3 min, then resuspended in a single cell suspension in DMEM media for sci-Plex processing. Single-cell transcriptome sequencing was performed using the Chromium Next GEM Single Cell 3′ Kit (10× Genomics, Hamburg, Germany, 1000268). Approximately 5000 live cells from each sample were loaded onto a Chromium Next GEM Chip G (10× Genomics, #1000127) and processed in a Chromium Controller device (10× Genomics, Hamburg, Germany, #120223;120246). The single-cell transcriptome library was then generated exactly according to the manufacturer’s guidelines. Finally, the library was sequenced on a NextSeq2000 sequencer (Illumina, Berlin, Germany) by loading a concentration of 650 pM and performing on-board denaturation on a NextSeq 1000/2000 P2 Flowcell (100 cycles) (Illumina, Berlin, Germany, 20100987). This resulted in 116,302,317, 127,497,705, 93,475,288, and 149,278,971 reads per sample. The following sequencing parameters were used: read 1: 28 cycles (16 bp barcode, 12 bp UMI); index 1: 10 cycles; index 2: 10 cycles; read 2: 90 cycles (insert).

### 2.11. scRNAseq Data Analyses

The raw sequencing data were processed using DragenSingleCellRna (v3.8.4) with the reference genome hg38-noalt-with-decoy and converted to FASTQ format using BCLConvert (v3.8.4). The scRNA-seq data were analyzed using R package SoupX (v1.6.2), DropletUtils (v1.22.0), DoubletFinder (v2.0.4), and Seurat (v5.2.1). Empty cells and droplets were removed. Low-quality cells were filtered based on mitochondrial gene content (>10%) and feature counts (<200 genes per cell). Batch correction and dataset integration were performed using Canonical Correlation Analysis (CCA) Integration in Seurat. Cells were annotated by integrating with both Azimuth Human Fetus (https://azimuth.hubmapconsortium.org/references/human_fetus (accessed on 17 June 2025) and UCSC Retinal Organoid (PMID: 32946783, https://cells.ucsc.edu/?ds=roska-retina-atlas+organoid (accessed on 17 June 2025) [24], ensuring consistency across datasets. Cell type labels were reconciled based on the reference annotations. Pathway enrichment was assessed using R package escape (v2.2.3). Single-sample Gene Set Enrichment Analysis (ssGSEA) was computed using curated gene sets from msigdbr (v7.5.1). Data visualization was performed using ggplot2 (v3.5.1) and pheatmap (v1.0.12). All computations were carried out in R (v4.3.3) on Ubuntu 24.04.2 LTS.

### 2.12. Statistical Analysis

All quantitative experiments were performed with biological replicates from at least three independent differentiation batches of organoids per condition. Data are presented as mean ± standard error of the mean (SEM). Statistical analyses were conducted using unpaired two-tailed Student’s *t*-tests for comparisons between two groups (e.g., control vs. *PRPF31*-ROs) at given time points. Morphological measurements (such as outer segment length) in control vs. *PRPF31* organoids were compared by unpaired *t*-test. Similarly, gene expression levels determined by qPCR were analyzed by comparing fold change values between groups with a *t*-test. Normality of data distributions and homogeneity of variances were evaluated (using Shapiro–Wilk and Levene’s tests, respectively) to validate the use of parametric tests; when these assumptions were not met, a non-parametric Mann–Whitney test was planned. Statistical significance was defined at *p* < 0.05. Significance levels in figures are indicated with asterisks (* *p* < 0.05; ** *p* < 0.01; *** *p* < 0.001; **** *p* < 0.0001), and all hypothesis tests were two-tailed. All statistical computations for imaging and qPCR were performed using GraphPad Prism 9 (GraphPad Software, San Diego, CA, USA). For single-cell RNA-seq analyses, differentially expressed genes between conditions were identified using the Wilcoxon rank-sum test in Seurat with Bonferroni-adjusted *p* values; genes with adjusted *p* < 0.05 and substantial fold change were considered significantly different.

### 2.13. Data Availability

The dataset we generated for these studies is available through the NCBI GEO accession GSE307923 (https://www.ncbi.nlm.nih.gov/geo/query/acc.cgi?acc=GSE307923 (accessed on 17 June 2025).

## 3. Results

### 3.1. Generation and Characterization of ROs

**Figure 1:**
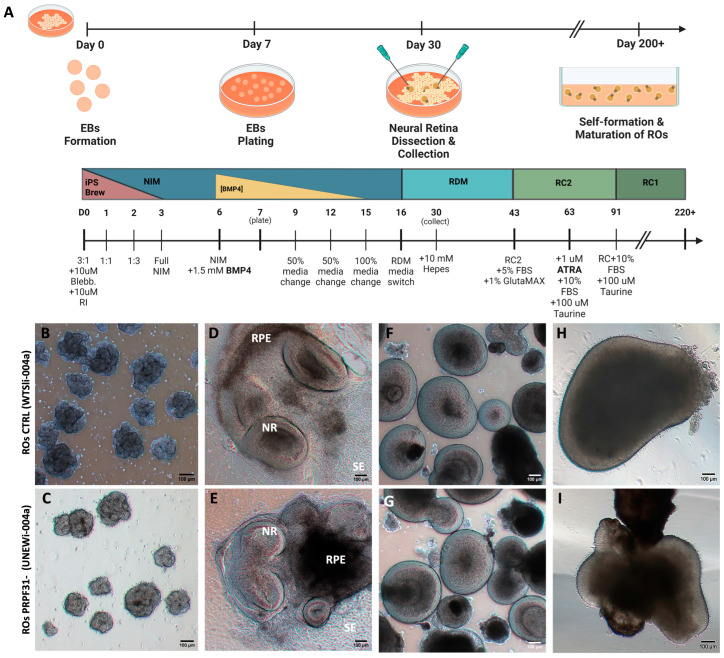
Formation and long-term culture of 3D retinal organoids (ROs): (**A**) Schematic representation of the stepwise retinal differentiation protocol from hiPSCs. (**B**–**I**) Phase-contrast images showing RO progressive differentiation over ~280 days in vitro. (**B**,**C**) Embryoid bodies (EBs) (day 7) formed from hiPSC colonies. (**D**,**E**) By day 30, optic vesicle-like structures with distinct neural retina (NR) and retinal pigment epithelium (RPE) domains emerge. (**F**,**G**) Isolated NR domains self-organize into optic cup-like 3D organoids that continue maturing in suspension (day 90). (**H**,**I**) Late-stage organoids (week 35) exhibit a thick outer layer rich in developing photoreceptors. Abbreviations: EB: embryoid bodies, SE: surface ectoderm, NR: neural retina, RPE: retinal pigment epithelium progenitor zone (Scale bar = 100 μm).

The iPSCs used in this study included a healthy control line and a line harboring the *PRPF31* c.1115_1125del11 mutation, which is associated with autosomal dominant RP (adRP) type 11 [25]. Both iPSC lines showed typical pluripotent stem cell morphology presenting tightly packed colonies and defined borders (Figure S1A,B) [26]. Immunofluorescence showed a strong nuclear expression of core pluripotency markers OCT4, SOX2, and NANOG in both WT and *PRPF31* iPSCs (Figure S1C–H). Quantitative analysis through flow cytometry showed that >90% of cells were positive for TRA-1-60 (Figure S2A–F). PCR amplification verified the presence of the *PRPF31* mutation in the RP11 line, but not in the control line (Figure S2G), and immunostaining for *PRPF31* protein at D60 revealed clear nuclear expression in control ROs but a significant ~5-fold reduction in PRPF31+ cells in *PRPF31*-ROs (*p* < 0.01) (Figure S3). Then, both healthy control and *PRPF31*-RP11 hiPSCs were differentiated into ROs (Figure 1A), adapting previously established protocols [22,23]. Briefly, iPSC colonies were cultured to 70–80% confluency before neural induction, transitioning through a stepwise medium change. By day 7, embryoid bodies (EBs) (Figure 1B,C) were plated on Matrigel-coated dishes to promote retinal progenitor development. Optic vesicle-like structures emerging by day 30 (Figure 1D,E), were manually isolated, and maintained in suspension (Figure 1F,G). By day 250, ROs from both lines showed retinal maturation morphology (Figure 1H,I), with no apparent culture complications or delayed culture progression.

### 3.2. Early Effects of the PRPF31 Mutation on ROs Development

**Figure 2:**
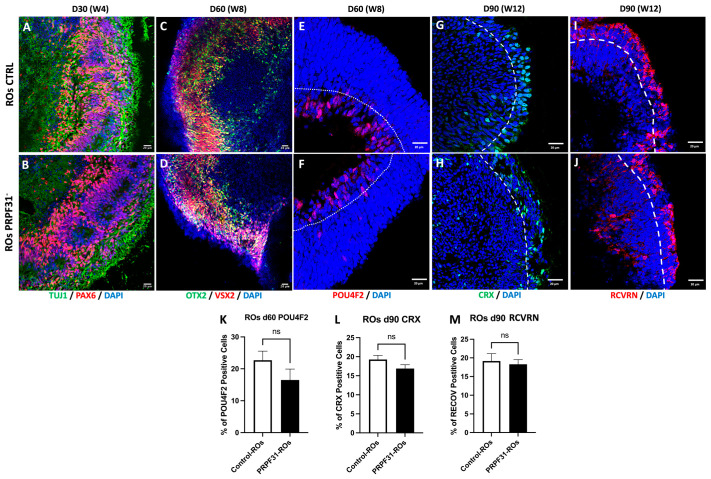
Early retinal differentiation in control vs. *PRPF31*-ROs: Immunofluorescence images of ROs at early time points. (**A**,**B**) Staining for PAX6 (retinal progenitor marker, red) and TUJ1 (early neuron marker, green) in day 30 control (**A**) and *PRPF31*-ROs (**B**). Both conditions show robust PAX6^+^ and TUJ1^+^ populations, indicating similar initial retinal neurogenesis. (**C**–**F**) OTX2 (photoreceptor/bipolar progenitor marker, green) and VSX2 (retinal progenitor/bipolar marker, red) labeling in day 60 control (**C**) vs. *PRPF31*-ROs (**D**) reveal layered OTX2^+^/VSX2^+^ cells in controls and *PRPF31*-ROs. POU4F2 (retinal ganglion cell marker, red) staining in day 60 control (**E**) vs. *PRPF31*-ROs (**F**) shows RGCs presence in both conditions. (**G**,**H**) CRX (photoreceptor progenitor marker, red) and (**I**,**J**) RCVRN (mature photoreceptor marker, green) immunolabeling on day 90 Control (**G**,**I**) and *PRPF31*-ROs (**H**,**J**) demonstrate numerous CRX^+^ and RCVRN^+^ photoreceptor cells in both; however, in *PRPF31*-ROs these cells are disorganized and fail to form a distinct ONL. (**K**) Quantification of RGCs population at day 60. The bar graph indicates no significant difference in the number of POU4F2 positive cells between conditions. (**L**,**M**) Quantification of photoreceptor populations at day 90. Bar graphs show the percentage of CRX+ cells (**K**) and RCVRN^+^ cells (**L**) out of total DAPI^+^ cells in control vs. *PRPF31*-ROs. No significant differences are observed in these proportions between conditions, indicating that overall photoreceptor cell yield is similar despite structural disorganization. All data are presented as mean ± SEM from *n* = 5 organoids per condition, across at least three independent differentiation experiments. White dotted line: ganglion cell layer (GCL); White dashed line: outer nuclear layer (ONL).

To assess retinal cell fate specification and investigate whether mutations in the *PRPF31* gene impact the differentiation and organization of retinal cell types within our successfully generated ROs, the expression of key retinal markers was analyzed at several early developmental time points (D30, D60, and D90). At D30, early retinal neurogenesis was evaluated by immunofluorescence using TUJ1 and PAX6 (early neural and eye field development markers, respectively) (Figure 2A,B) [27]. Both control and *PRPF31* organoids formed a neuroepithelial “optic cup” stage and showed a robust TUJ1^+^ and PAX6^+^ immunoreactivity without apparent differences. On day 60, immunofluorescence for OTX2 and VSX2 (retinal progenitor and bipolar cell markers) [28,29] revealed that both control and *PRPF31*-ROs showed a layered distribution of OTX2+ and VSX2+ cells (Figure 2C,D). Additionally, POU4F2 staining indicated proper differentiation of RGCs in both control and *PRPF31*-ROs, localized in the inner core of the RO—the ganglion cell layer (GCL) (Figure 2E,F). Quantification of the number of POU4F2^+^ cells in ROs at 60 days of maturation showed a slight reduction in RGCs in *PRPF31*-ROs but with no significant difference (Figure 2K). At D90, differentiation and maturation of photoreceptor cells were assessed by immunolabeling for CRX (photoreceptor progenitor marker) and Recoverin (RCVRN, photoreceptor marker) (Figure 2G–J) [30,31]. Both control and *PRPF31*-ROs exhibited clear CRX+ and RCVRN+ staining, indicative of successful photoreceptor differentiation. Control ROs staining (Figure 2G,I) exhibits a continuous outer layer of CRX^+^ nuclei (Figure 2G) and RCVRN^+^ cells (Figure 2I) in the same domain, consistent with early photoreceptor nuclei occupying the developing outer nuclear layer (ONL). *PRPF31*-ROs staining (Figure 2H,J) also shows CRX^+^ (Figure 2H) and RCVRN^+^ (Figure 2J) cells at the periphery, but the signal appears more discontinuous. To quantitatively assess these observations, the proportion of CRX^+^ and RCVRN^+^ cells to the total number of DAPI^+^ cells was analyzed (Figure 2L,M). Despite the structural differences noted, quantitative analyses revealed no statistically significant differences in the number of CRX^+^ and RCVRN^+^ cells between control and *PRPF31*-ROs. This suggests that, while early differentiation events occurred similarly in both conditions, potential impairments in retinal cell specification and differentiation might become more apparent at later stages.

### 3.3. The PRPF31 Mutation Leads to Photoreceptor Disorganization and Impaired Marker Expression in ROs

**Figure 3:**
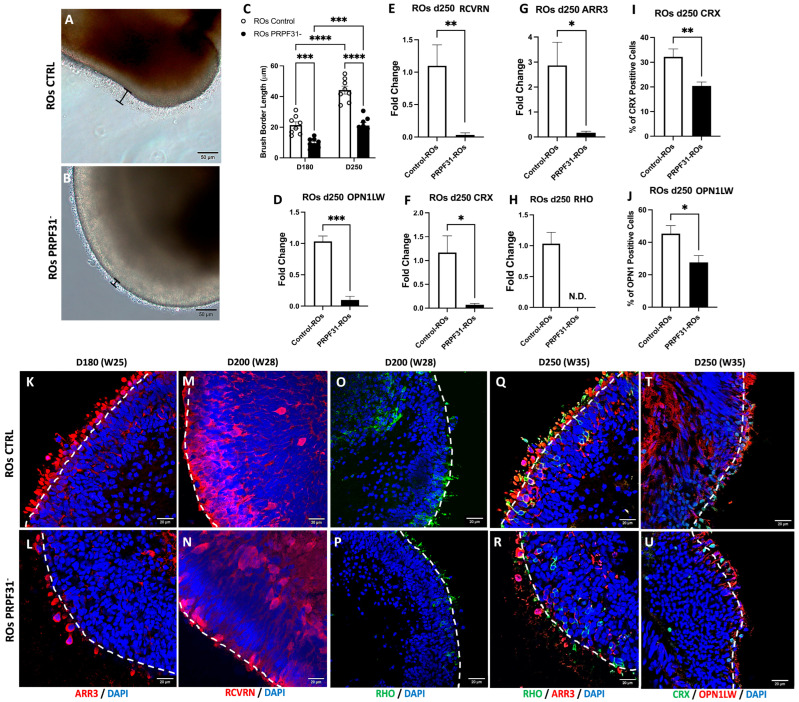
*PRPF31* mutation disrupts photoreceptor layer structure and gene expression in late-stage retinal organoids: (**A**,**B**) Brightfield images of the outer surface (“brush border” marked in black brackets) in day 250 control and *PRPF31*-ROs. Control ROs (**A**) exhibit a well-defined brush border, whereas *PRPF31*-ROs (**B**) display a significantly shorter and disorganized brush border. (**C**) Quantification of brush border thickness at day 180 and day 250, showing a significant reduction in *PRPF31*-ROs compared to controls (**** *p* < 0.0001, Two-way ANOVA). Error bars represent standard error of the mean (SEM), *n* = 8. (**D**–**H**) mRNA expression of key photoreceptor genes in day 250 ROs by RT–qPCR. *PRPF31*-ROs exhibit dramatically lower expression of *RCVRN* (**D**), ARR3 (**E**), *OPN1LW* (cone opsins), (**F**) CRX, (**G**) and *RHO* (**H**) relative to controls. Data are normalized to control expression levels (** *p* < 0.01; *** *p* < 0.001; N.D., not detected, *n* = 5). (**I**,**J**) Quantification of differentiated photoreceptors at day 250. Bar graphs show the number of CRX^+^ cells (**I**) and OPNLW1^+^ cone cells (**J**) in control vs. *PRPF31*-ROs. *PRPF31*-ROs have significantly fewer CRX^+^ precursors (* *p* < 0.05) and OPN1LW^+^ cones (** *p* < 0.01) than controls, *n* = 10. (**K**–**R**) Immunofluorescence images of photoreceptor markers in organoid sections. (**K**,**L**) ARR3^+^ cone photoreceptors at day 180 in control vs. *PRPF31*-ROs: cones are abundant with elongated morphology in controls (**K**) but are reduced in number and visibly stunted/disrupted in *PRPF31*-ROs (**L**). (**M**,**N**) RCVRN staining at day 200 highlights both rod and cone photoreceptors; control organoids (**M**) show dense photoreceptor layers, whereas *PRPF31*-ROs (**N**) have sparse photoreceptor labeling, confirming loss of photoreceptor cells. (**O**–**R**) Day 200 and 250 immunostaining for RHO (rods, green) (**O**,**P**) and ARR3 (cones, red) (**Q**,**R**) in control vs. *PRPF31*-ROs: control (**O**,**Q**) retains organized rod and cone layers, while *PRPF31*-ROs (**P**,**R**) show disorganized, faint rod/cone signals consistent with photoreceptor degeneration. (**T**,**U**) CRX (photoreceptor progenitor, green) and OPN1LW (L/M-cone opsin, red) staining at day 250; control (**T**) exhibits many CRX^+^ precursors and mature cones, whereas *PRPF31*-Ros (**U**) have fewer CRX^+^ cells and OPN1LW^+^ cones. Data are presented as mean ± SEM from *n* = 10 organoids per condition, across at least three independent differentiation experiments. White dashed line: outer limiting membrane (OLM).

To assess the impact of *PRPF31* mutation on RO development and photoreceptor maturation, we examined the morphology and expression of key photoreceptor markers at later time points. Firstly, we examined the RO brush border length after 180 d and 250 d of differentiation, which is indicative of the presence of mature photoreceptors. Brightfield imaging revealed that control ROs at both stages developed a well-defined brush border, maintaining the expected length of about 20 and 40 μm, respectively [32]; in contrast to *PRPF31*-ROs, who showed a shorter and disorganized brush border (Figure 3A,B). Quantification confirmed a statistically significant reduction in brush border length in *PRPF31*-ROs as compared to controls (*p* < 0.0001) at 180 and 250 d (Figure 3C). Correspondingly, *PRPF31*-ROs showed significantly reduced mRNA expression of *ARR3* (a cone photoreceptor marker) [33], *RCVRN*, (essential for photoreceptor signaling) [34,35], *CRX* (key marker and regulator of photoreceptor identity) [36,37], *OPN1LW* (a cone opsin) [38], and Rhodopsin (*RHO*, a rod-specific protein) [38] compared to controls (Figure 3D–H). Notably, *RHO* mRNA was undetectable in *PRPF31*-ROs. Immunofluorescence further corroborated these findings, showing decreased expression of photoreceptor markers at different differentiation stages (Figure 3K–R,T,U). By day 180, control ROs staining displayed a robust band of ARR3^+^ cone photoreceptors with elongated, mature morphology extruding the organoid outer limiting membrane (OLM), whereas *PRPF31*-ROs had fewer ARR3^+^ cells that appeared stunted and disorganized (Figure 3K,L). At day 200, RCVRN staining revealed a dense, multilayered photoreceptor region in control organoids, while *PRPF31*-ROs showed only sparse RCVRN^+^ cells, probably due to the degeneration of photoreceptors or their immature state (Figure 3M,N). At D200 and D250, control organoids retained well-organized outer retinal layers: RHO^+^ rods and ARR3^+^ cones formed contiguous photoreceptor bands (Figure 3O,Q). In contrast, *PRPF31*-ROs staining exhibited only weak, diffuse RHO and ARR3 signals (Figure 3P,R), consistent with photoreceptor degeneration. Likewise, controls at day 250 had abundant CRX^+^ precursors and mature OPN1LW^+^ cones arranged in a distinct outer nuclear layer (Figure 3T), whereas *PRPF31*-ROs contained fewer CRX^+^ and OPN1LW^+^ cells with no defined ONL (Figure 3U). Quantification confirmed these observations: the number of CRX^+^ progenitors and OPN1LW^+^ cones was significantly lower in *PRPF31*-ROs (*p* < 0.01 and *p* < 0.05, respectively) (Figure 3I,J). To assess whether late-stage ROs develop necrotic cores, apoptosis was evaluated by cleaved Caspase-3 (CAS3) [39] immunostaining at D250 (Supplementary Figure S4). Quantification revealed no significant difference in CAS3^+^ cells within the necrotic core region between control and *PRPF31*-ROs. In contrast, CAS3^+^ cells within the inner retinal layer (INL) were significantly increased in *PRPF31*-ROs, corresponding to a 2.6-fold increase compared to controls, indicating localized apoptotic activity rather than widespread necrosis (Supplementary Figure S4). Given the intricate cellular interactions within the retinal tissue, we questioned whether *PRPF31* impairment might also affect the functionality of other key retinal neurons, in particular the RGCs, which play a crucial role in transmitting visual information to the brain [40].

### 3.4. Impaired Electrophysiological Responses in PRPF31-ROs

**Figure 4:**
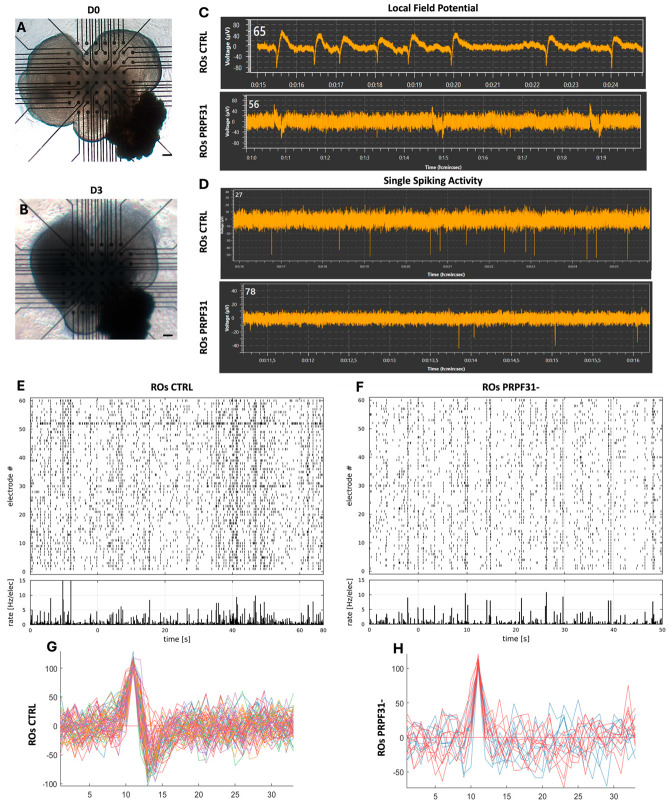
*PRPF31* deficiency impairs spontaneous retinal ganglion cell (RGC) activity in organoids: (**A**,**B**) Brightfield images of retinal organoids positioned on multi-electrode array (MEA) chips at Day 0 and after 3 days (start of recording), demonstrating stable organoid placement over time. (**C**,**D**) Representative recordings of local field potentials (**C**) and single-unit spiking activity (**D**) from control and *PRPF31*-ROs, demonstrating spontaneous neural activity captured via MEA analysis. (**E**,**F**) Raster plots of spontaneous spiking activity recorded across MEA electrodes in control (**E**) and *PRPF31*-ROs (**F**). Each dot represents a spike event at a specific electrode and time point. The accompanying histograms display the overall firing rate across electrodes. Control organoids exhibit robust, widespread spiking across multiple channels, whereas *PRPF31*-ROs show reduced spike density and lower overall firing rates, indicating impaired spontaneous neuronal network activity. (**G**,**H**) Superimposed spike waveforms from control (**G**) and *PRPF31*-ROs (**H**) reveal overall preserved spiking patterns, although *PRPF31*-ROs display a reduced number of active channels and diminished spike amplitudes. Recordings were conducted on 10 ROs per condition, out of which *n =* 3 showed consistent spontaneous activity.

Spontaneous neural activity is a hallmark of functional retinal circuits and plays a crucial role in the early maturation of visual processing networks [24,41]. Therefore, to investigate the functional consequences of the *PRPF31* mutation on retinal neuronal network activity, we performed MEA recordings on day 60 (D60) ROs derived from both control and *PRPF31*- hiPSCs. Brightfield imaging confirmed stable attachment of the organoids onto the MEA grid from D0 to D3, ensuring consistent recording conditions (Figure 4A,B). Immunofluorescence analysis for the RGC marker POU4F2 revealed the presence of RGC populations in both groups (Figure 2E,F,K). In control ROs, representative local field potential (LFP) traces showed regular oscillatory fluctuations, reflecting robust spontaneous synaptic activity (Figure 4C). Correspondingly, high-frequency single-unit spikes were detected throughout the recording in control ROs (Figure 4D). By contrast, the LFP recordings from *PRPF31*-ROs showed only small, infrequent deflections (Figure 4C), and the single-unit spiking traces showed far fewer discernible spikes (Figure 4D). Network-level analyses using raster plots on control ROs showed broadly distributed spikes across many electrodes, with frequent synchronized firing events (Figure 4E). The accompanying per-electrode firing rate histogram showed high overall activity, consistent with robust and well-coordinated spontaneous network bursts. In contrast, *PRPF31*-ROs displayed a sparse scattering of spike events across the electrodes, with many channels registering little to no activity (Figure 4F). Additional raster plots of MEA activity from two additional ROs are shown in Supplementary Figure S5. In addition, superimposed spike traces from control ROs revealed consistent, high-amplitude waveforms across multiple recording sites (Figure 4G). In contrast, *PRPF31*-ROs recordings showed a reduced amplitude in their spike waveforms and a notable decrease in the number of electrodes registering spontaneous activity (Figure 4H), suggesting impaired network connectivity and reduced RGC excitability. The reduction in spike amplitude, firing rate, and network synchronization in *PRPF31*-ROs reflects a deficit in the formation or maintenance of excitatory functional circuits.

### 3.5. scRNAseq Revealed Early Müller Glia Marker Expression and Late Photoreceptor Transcriptomic Collapse in PRPF31-ROs

**Figure 5:**
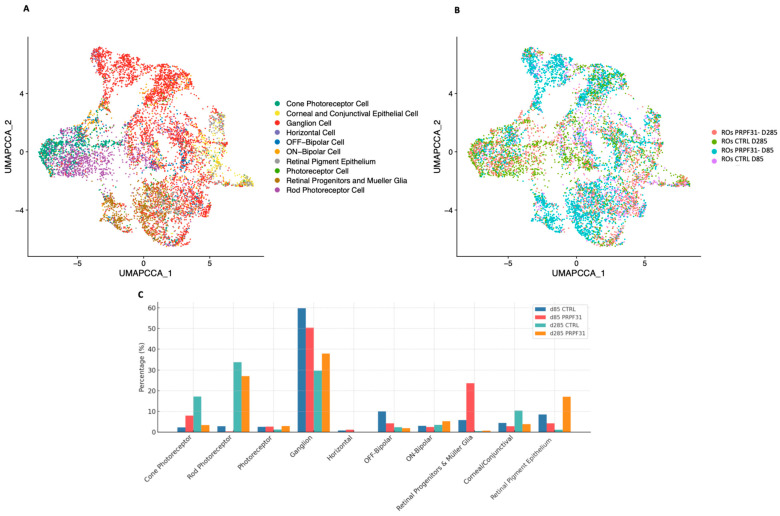
Single-cell RNAseq of control and *PRPF31*-ROs reveals cell population shifts: (**A**) UMAP embedding of all cells from early (D85) and late (D285) ROs across both conditions, colored by annotated retinal cell type. All major retinal lineages were identified, including cones, rods, ganglion cells, bipolar subtypes, horizontal cells, Müller glia/progenitors, corneal/conjunctival-like cells and retinal pigment epithelium. (**B**) UMAP plot of the same dataset colored by sample identity, illustrating the distribution of cells from each condition (control vs. *PRPF31*) and differentiation stage (D85, D285). (**C**) Bar plot of relative cell-type proportions across conditions and stages, showing high Müller glia gene expression and cone enrichment in *PRPF31* mutant ROs at D85, followed by severe cone gene expression and moderate rod reduction at D285.

To evaluate whether our hiPSC-derived ROs accurately recapitulate the cellular complexity and organization of the adult human retina, and to assess the molecular impact of *PRPF31* haploinsufficiency in our investigated patient line, scRNA-seq analysis was conducted on early (d85) and late (d285) stage ROs using the 10× Genomics platform. The analysis identified 10 distinct cell population clusters (Figure 5A,B), which were annotated by integrating our data with the UCSC Human Retina Atlas reference database [24] and Azimuth human fetus data [42]. This comprehensive map of the retinal cell types revealed the establishment of all major retinal cell lineages in organoids, including photoreceptors, RGCs, bipolar cells, horizontal cells and Müller glia. Minor proliferative and progenitor cell clusters were also present, reflecting ongoing retinal development in the organoids. The presence of this broad spectrum of retinal cell types demonstrates that our generated ROs undergo an in vitro retinogenesis that mirrors normal human retinal development [43]. Cell-type-specific gene annotation signatures are shown in Supplementary Table S4. Uniform Manifold Approximation and Projection (UMAP) plots clearly demonstrated distinct and well-organized clustering patterns corresponding to various retinal cell types in all the processed samples (Supplementary Figure S6A–D). The cellular heterogeneity and composition of the ROs closely resembled the human native retina and is comparable to published single-cell ROs datasets (Supplementary Figure S7).

Having validated the composition of our organoids, we next compared control vs. *PRPF31*-ROs to investigate the impact of the investigated *PRPF31* mutation on retinal cell dynamics and identify disease-related changes in cell populations and gene expression during organoid differentiation (Figure 5C). At D85, *PRPF31*-ROs data showed a high increase in Müller glia/progenitor cell population (23.6% vs. 5.8% in controls; ~4.1-fold increase), together with a modest reduction in RGCs (50.4% vs. 59.8% in controls; ~1.2-fold decrease). The photoreceptor clusters are already disproportionate at this stage: cones are enriched in mutants (8.0% vs. 2.3% in controls; ~3.5-fold increase), whereas rods are nearly absent (0.3% vs. 2.8% in controls; ~9-fold decrease) (Figure 5C). At D285, the cellular consequences of the *PRPF31*-ROs consolidated into a photoreceptor-dominant deficit (Figure 5C and Figure S6). Control ROs data showed the expected late pattern, with rods (33.8%) and cones (17.2%) forming the largest neuronal classes, whereas *PRPF31*-ROs data revealed a severe decrease in cones (3.4%; ~5-fold decrease vs. controls) and a moderate reduction in rods (27.1%; ~1.3-fold decrease). In contrast, the RGC population was proportionally higher in mutants (37.9% vs. 29.7% in controls; ~1.3-fold increase). Müller glia/progenitors, which were over-represented at day 85, were low in both groups at day 285 (0.7% vs. 0.5% in controls). Additionally, although the RPE domain was largely removed during early optic vesicle isolation (D30) to enrich for neural retina, a small population of RPE cells was still detected and annotated, showing a rise in *PRPF31*-ROs at late stage (17.1% vs. 1.2% in controls).

### 3.6. Cell-Type-Specific Molecular Responses to the PRPF31 Mutation in ROs Drive Early Müller Glial Activation and Progressive Retinal Degeneration

**Figure 6:**
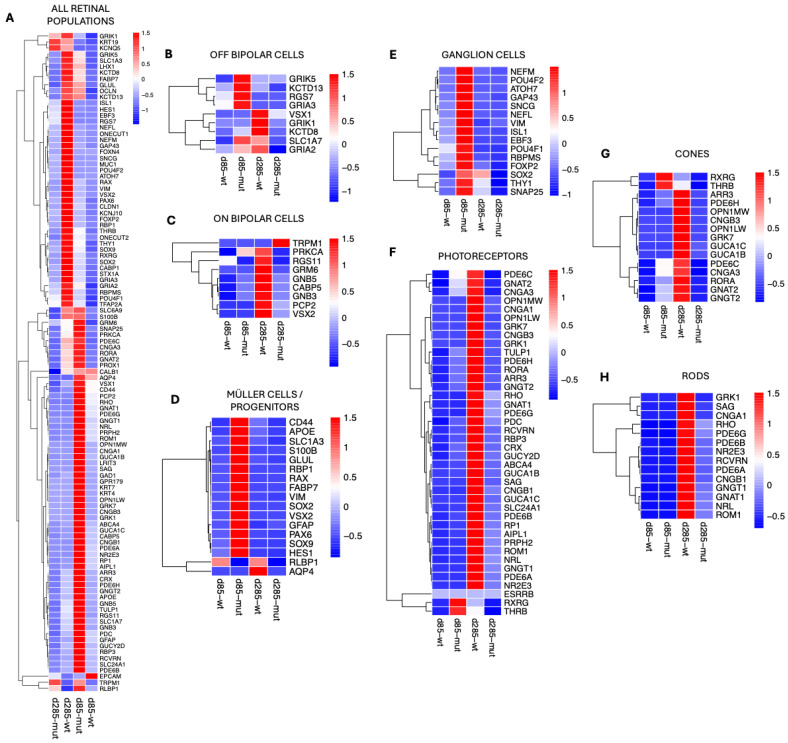
The *PRPF31* mutation induces cell-type-specific transcriptional dysregulation in retinal organoids: (**A**) Heatmap of all significantly differentially expressed genes (DEGs) across retinal cell types, highlighting broad upregulation of stress and gliosis markers and downregulation of phototransduction genes in *PRPF31*-ROs. (**B**–**H**) Cell-type-specific DEG heatmaps: (**B**) OFF-bipolar cells—mutants at day 85 transiently upregulate kainate/glutamate receptor-associated genes (*GRIK5*, *RGS7*, *KCTD13*, *GRIA3*, *SLC1A7*), while controls maintain robust *VSX1* and *GRIK1* expression at day 285. (**C**) ON-bipolar cells—controls show maturation with *GRM6*, *PCP2*, *PRKCA*, *CABP5*, and *VSX2* expression at day 285, whereas mutants display decrease expression of these markers and aberrant upregulation of TRPM1. (**D**) Müller glia—mutants at day 85 show strong upregulation of gliosis and progenitor/stress markers (*GFAP*, *VIM*, *SOX2*, *PAX6*, *S100B*, *APOE*, *FABP7*), whereas controls express homeostatic regulators (*RLBP1*, *AQP4*). (**E**) RGCs—*PRPF31*-ROs at day 85 upregulate multiple identity and axonogenesis genes (*RBPMS*, *POU4F1/2*, *THY1*, *NEFL/M*, *GAP43*, *ISL1*, *EBF3*, *ATOH7*, *SNAP25*, *FOXP2*), but by day 285 these markers decline compared to controls. (**F**) Photoreceptors—global reduction in phototransduction-related genes in mutants compared to controls. (**G**) Cone photoreceptors—controls maintain robust *ARR3*, *GNAT2*, *OPN1MW/LW*, *PDE6C/H*, *CNGA3*, *CNGB3*, and *RORA* expression at day 285; mutants transiently upregulate *RXRG* and *THRB* at day 85 but fail to maintain cone-specific gene expression by day 285. (**H**) Rod photoreceptors—controls at day 285 show coordinated expression of *RHO*, *GNAT1*, *SAG*, *PDE6A/B/G*, *CNGA1*, *CNGB1*, *NRL*, *NR2E3*, and *RCVRN*, whereas mutants fail to sustain these markers.

To elucidate the molecular mechanisms driving retinal degeneration caused by the *PRPF31* mutation in ROs, our analysis focused next on the identification of gene expression differences across retinal cell populations in control versus *PRPF31*-ROs. To accomplish this, gene expression profiles were generated from the scRNAseq clusters (Figure 6). Heatmaps displaying global gene expression patterns show variations in gene expression between control and *PRPF31*-ROs across different retinal populations (Supplementary Figure S8). We then proceeded to systematically identify differentially expressed genes (DEGs) in control and *PRPF31*-ROs, within each retinal cell population (Figure 6A–H). The identified DEGs were normalized to account for technical and biological variation.

At day 85, *PRPF31*-ROs showed a transient upregulation of several OFF-bipolar cell genes—including *GRIK5* [44,45], *RGS7* [46], *KCTD13* [47], and *GRIA3* [48]. However, by late differentiation (day 285), OFF-bipolar cell markers were reduced in *PRPF31*-ROs (Figure 6B). Control organoids maintained high expression of key ON-bipolar components (e.g., *GRM6* [49], *PCP2* [50], *PRKCA* [51], *CABP5* [52], *RGS11* [53], *GNB3*, *GNB5* [54], *VSX2* [29]), whereas these were downregulated in *PRPF31*-ROs by day 285. Notably, the ON-bipolar transduction channel *TRPM1* [55,56] was abnormally upregulated in D285 *PRPF31*-Ros, even as other ON-bipolar signals (like mGluR6 signaling via GRM6) were lost (Figure 6C).

Müller cells mounted one of the earliest and most robust responses to the *PRPF31* mutation. By day 85, *PRPF31*-ROs data showed strong upregulation of glial fibrillary acidic protein (*GFAP*) [57,58], along with other gliosis markers such as *VIM* (vimentin) [58,59], and the glutamate-buffering enzymes *SLC1A3* (*GLAST*) [60] and *GLUL* (glutamine synthetase) [61] (Figure 6D). Consistently, Müller/progenitor cells in the mutant ROs showed elevated expression of pro-inflammatory or stress-associated genes, including *APOE* [62], *S100B* [63], and *FABP7* [64]. At day 85, DEG analysis in RGCs also showed higher expression of synaptic and axonogenesis-related genes (e.g., *SNAP25* [65,66] and *GAP43* [67,68]) relative to controls (Figure 6E). They also upregulated general RGC identity markers like *RBPMS* [69,70], *POU4F1/POU4F2* [71,72], (*BRN3A/3B* transcription factors), and *THY1* [69,73], as well as neuronal cytoskeletal components (*NEFL*, *NEFM* [69,74] encoding neurofilament proteins). Meanwhile, certain RGC maintenance factors were dysregulated over time—notably, the transcription factor *SOX2* [75], which was transiently higher in *PRPF31* RGCs at D85 but declined thereafter. By day 285, *PRPF31*-ROs had fewer RGCs overall and much lower expression of RGC-specific genes compared to control ROs. In contrast, age-matched wild-type RGCs retained higher *SOX2* and other homeostatic markers at late stages.

Photoreceptor populations showed dramatic genotype-dependent differences by late differentiation (Figure 6F). For instance, control cones expressed robust levels of cone opsins (*OPN1MW/LW* for medium/long-wavelength opsins), cone transducin (*GNAT2*) [76], cone arrestin (*ARR3*), phosphodiesterase subunits (*PDE6C*, *PDE6H*) [77,78], cyclic nucleotide-gated channel subunits (*CNGA3*, *CNGB3*) [79,80,81], other cone-specific proteins like *GUCA1B/1C* [82], *GNGT2* [83], *GRK7*, and the cone nuclear receptor *RORA* [84] (Figure 6G). In contrast, DEG analysis on *PRPF31*-ROs cones at D285 showed downregulation of nearly all cone opsin and phototransduction markers (Figure 6G). Intriguingly, at an earlier stage (D85) the mutant cones transiently upregulated certain cone developmental regulators—notably the nuclear hormone receptors *RXRγ* [85] (retinoid X receptor gamma) and *TRβ2* [86,87,88] (thyroid hormone receptor beta-2) (Figure 6G).

Rod photoreceptors were even more severely affected by the *PRPF31* mutation. In healthy ROs at D285, rods exhibited robust expression of canonical rod phototransduction markers—including *RHO* [89], rod transducin *GNAT1* [90], rod arrestin *SAG* [90], the α and β subunits of the rod cGMP-gated channel (*CNGA1*, *CNGB1*) [79], the rod phosphodiesterase subunits *PDE6A*, *PDE6B*, *PDE6G*, [77,91], and the rod-specific G-protein *GNGT1* [90] (Figure 6H). Additionally, control rods showed strong expression of rod differentiation transcription factors *NRL* and *NR2E3*, as well as calcium-binding protein *RCVRN* [34,35]—a coordinated gene expression profile indicating normal rod maturation and phototransduction capacity. In *PRPF31*-RO derived rods, however, these photoreceptor transcripts failed to be maintained. By day 285, all of the aforementioned rod-specific genes were dramatically downregulated in mutant organoids relative to controls, consistent with widespread rod photoreceptor degeneration (Figure 6H). In fact, the scRNAseq data are consistent with our previous molecular assays: for example, *RHO* qRT-PCR mRNA expression was undetectable by ~D250 in *PRPF31*-ROs, and immunostaining revealed a stark loss of RHO^+^ and ARR3^+^ cells in the *PRPF31*-ROs (Figure 3).

### 3.7. Pathway Enrichement on scRNAseq Data Revealed Alterated Pathways Involved in Phototransduction, Oxidative Stress, and Inflammation

**Figure 7:**
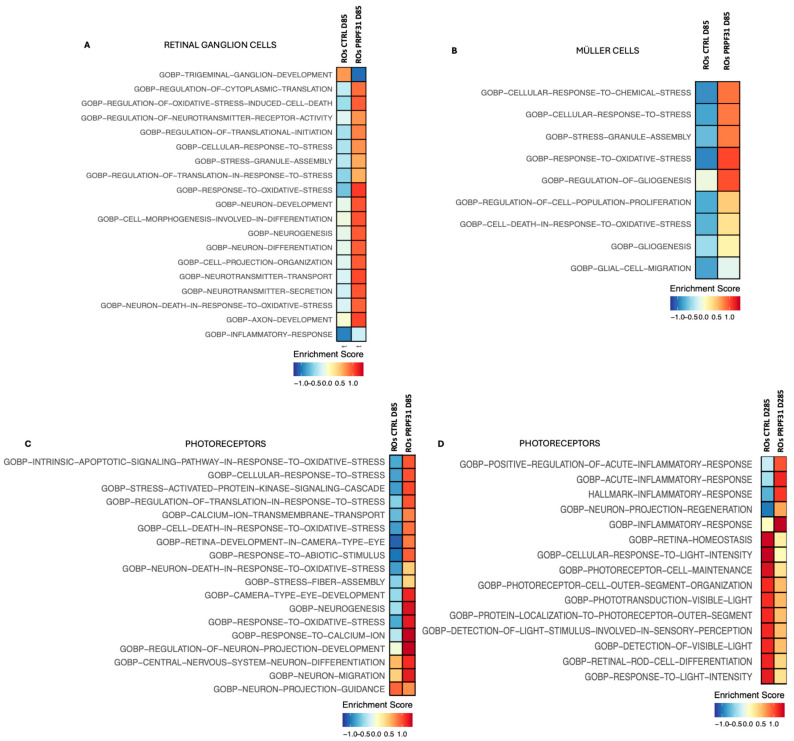
Enriched pathway analysis of differentially expressed genes in *PRPF31*-ROs: Gene ontology (GO) and pathway enrichment results for DEGs within specific retinal cell types, highlighting the biological processes most affected by the *PRPF31* mutation. (**A**) Top enriched pathways in *PRPF31*-ROs RGCs (d85-mut) at an early stage include oxidative stress response, regulation of neuron death, and axon development. (**B**) Müller cells in *PRPF31*-ROs (d85-mut) show enrichment of pathways related to response to chemical and oxidative stress, and gliogenesis. (**C**,**D**) Rod and cone photoreceptors in *PRPF31*-ROs on day 85, aside from oxidative stress signaling; phototransduction pathways remain largely intact early on (**C**). By day 285 (**D**), *PRPF31* photoreceptors show significant enrichment for inflammatory response pathways, alongside a downregulation of phototransduction and light detection pathways. Key downregulated processes include outer segment morphogenesis and retinal cell maintenance, reflecting the collapse of photoreceptor structure and function at end-stage.

Having characterized significant cellular and transcriptomic disturbances associated with the *PRPF31* mutation, we next aimed to identify the broader biological processes influenced by these transcriptional changes. To achieve this, we performed pathway enrichment analysis utilizing the significant DEGs identified across retinal cell populations in control and *PRPF31*-ROs (Figure 7). RGCs in *PRPF31*-ROs at D85 exhibited pathway enrichments predominantly associated with cellular stress, inflammatory responses, neurotransmission, neuron differentiation, and axon development (Figure 7A). At the early differentiation stage (D85), Müller glial cells in *PRPF31*-ROs showed significant enrichment of pathways related to cellular stress responses and gliosis (Figure 7B). Specifically, pathways such as cellular response to chemical stress, oxidative stress response, gliogenesis, and glial cell migration were activated. Late-stage (d285) Müller cells/progenitors and RGCs showed no major altered pathway between control and *PRPF31*-ROs. At D85 ROs, photoreceptor populations exhibited signs of stress, as suggested by enrichment of oxidative stress responses, although phototransduction pathways appeared intact at this time (Figure 7C). However, by the advanced differentiation stage (D285), photoreceptors data showed striking alterations in pathways associated with photoreceptor function and stress response (Figure 7D). Significantly downregulated pathways included phototransduction, detection of visible light, retinal rod cell differentiation, and photoreceptor maintenance. Concurrently, pathways associated with oxidative stress and apoptosis were highly enriched, underscoring severe functional impairment and progressive degeneration of rods. Additionally, pathways involved in inflammatory and acute inflammatory responses, as well as neuron projection regeneration, were activated (Figure 7D).

### 3.8. Upregulation of STAT3 and S100B in PRPF31-ROs

**Figure 8:**
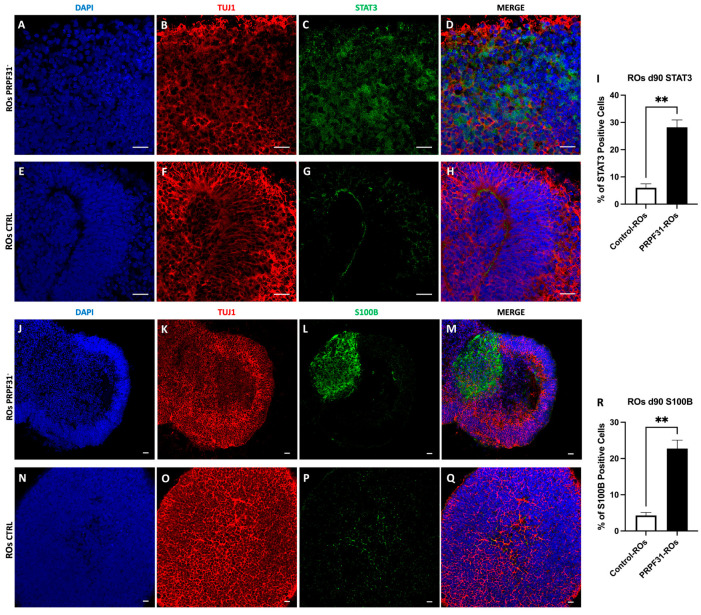
Immunofluorescence analysis of STAT3 and S100B in control and *PRPF31* retinal organoids. Immunostaining for STAT3 (green), S100B (green), TUJ1 (red; neuronal marker), and DAPI (blue; nuclei) in D85 retinal organoids (ROs). (**A**–**H**) STAT3 staining in *PRPF31*-ROs (**A**–**D**) shows widespread distribution compared with minimal and spatially restricted expression in control ROs (**E**–**H**). (**I**) Quantification of STAT3^+^ cells reveals a significant increase from ~6% in controls to ~28% in *PRPF31*-ROs (** *p* < 0.01; *n =* 5 organoids per condition). (**J**–**Q**) S100B immunoreactivity is sparse in control ROs (**N**–**Q**) but increased in *PRPF31*-ROs (**J**–**M**). (**R**) Quantification of S100B^+^ cells shows an increase from ~4% in controls to ~23% in *PRPF31*-ROs (** *p* < 0.01; *n =* 5 organoids per condition). TUJ1 staining demonstrates a well-organized neuronal plexiform network in control ROs, whereas *PRPF31*-ROs exhibit localized disruption in regions with elevated STAT3/S100B expression. Scale bars: 20 μm.

To further validate the scRNA-seq findings, we performed immunofluorescence analyses on D85 ROs using the gliotic and retinal distress markers STAT3 and S100B (Figure 8). In control ROs, STAT3 expression was minimal and spatially restricted, with sparse nuclear positivity observed in a small fraction of cells (~6%), predominantly localized at the inner retinal edge, consistent with a low basal level of physiological signaling and absence of overt gliosis (Figure 8E–I). Similarly, S100B immunoreactivity was limited to small, discrete cell clusters, accounting for approximately ~4% of total cells (Figure 8N–R). TUJ1 staining revealed a dense, continuous, and well-organized neuronal plexiform network throughout the neuroepithelial layers, reflecting preserved retinal cytoarchitecture (Figure 8F,O). In contrast, *PRPF31*-ROs staining displayed a widespread immunoreactivity of both STAT3^+^ and S100B^+^ cells (Figure 8A–D,J–M). STAT3^+^ cells were predominantly distributed within the inner retinal layers but extended into more outer regions in several areas (Figure 8C). The percentage of STAT3+ cells was significantly (4.7-fold) increased compared to control ROs, reaching ~28% of total cells (Figure 8I). The percentage of S100B+ cells was similarly elevated, with ~23% of cells positive in *PRPF31*-ROs, representing a 5.7-fold increase relative to controls (Figure 8R).

Notably, regions exhibiting strong STAT3 and S100B immunoreactivity frequently overlapped with areas of disrupted TUJ1^+^ neuronal networks, characterized by fragmentation of axonal bundles and gaps within the plexiform layers (Figure 8B,K).

## 4. Discussion

Using patient-derived RPE and ROs, Buskin et al. provided the first human evidence that impaired in vivo splicing is restricted to patient-derived retinal cells [24]. Through comprehensive transcriptome and biochemical analyses, they showed that impaired pre-mRNA splicing appears to be confined to splicing programs that affect RNA processing itself. These deficiencies are correlated with defective primary cilium morphology and features of photoreceptors degeneration and cell stress in patient-specific ROs. Another study by Rodrigues et al. used two RP patient iPSC lines and an isogenic control to demonstrate that *PRPF31*-mutated ROs recapitulate the human RP phenotype, and highlighted how the restoration of *PRPF31* expression by gene augmentation can reverse photoreceptor degeneration [21]. However, these studies have primarily focused on photoreceptor and RPE dysfunction at late disease stages, leaving early molecular events and the involvement of other retinal cell types poorly defined [13,20,21].

**Impaired photoreceptors at late-stage *PRPF31*-ROs**

We generated ROs from *PRPF31* RP11 patient and control hiPSCs and performed a series of morphological, functional, and single-cell transcriptomics profiling at early and late-stage ROs. We first confirmed that both the *PRPF31*- and control lines efficiently generated ROs and presented early progenitors to differentiated neurons aligning with prior studies [24,43]. This successful retinal differentiation of *PRPF31*-hiPSCs suggests that haploinsufficiency of this splicing factor does not overtly impede the early steps of retinogenesis [92]. By two months in culture (D60), *PRPF31*-ROs showed signs of disorganization of RGCs and photoreceptors without significant differences in total cell number, suggesting that the investigated *PRPF31* deficiency does not affect the production of photoreceptors early on but rather impacts their proper localization or maturation. Similar observations have been reported in other iPSC-based models of adRP, where *PRPF31*-ROs form the expected cell types yet display early disorganization or delayed maturation [23,25]. After approximately 6 months in vitro, *PRPF31*-ROs showed morphological and molecular defects in photoreceptors, reflecting a degenerative phenotype consistent with the classic RP11 pattern (rods followed by cones degeneration) and in recent *PRPF31* iPSC organoid models [21,92]. In addition, recent molecular analyses showed that *PRPF31* insufficiency leads to widespread mis-splicing and dysregulation of photoreceptor-specific transcripts [4,93], which likely compromises photoreceptor survival as the organoid ages. Defects in the photoreceptor ciliary structure have also been noted, such as shortened connecting cilia/outer segments in *PRPF31* mutant models [21], which dovetails with our observation of stunted outer segments. It should be noted that the temporal progression and subsequent degenerative phenotype observed in vitro might be affected by several technical elements during RO differentiation, rendering them completely different from the in vivo situation. In fact, the use of lineage specification and maturation molecules such as BMP4, retinoic acid, and Taurine, the simplified microenvironment in the culture dish, and the lack of vasculature to the organoids impose significant limitation sand could all affect normal and temporal cellular differentiation and maturation compared to in vivo [94,95].

**Reduced electrical activity in *PRPF31*-ROs**

Functional analyses using MEA recordings further reinforced the divergence between healthy and mutated ROs, as shown by a reduction in RGC activity, fewer active electrodes and lower overall spike rates in *PRPF31*-ROs. However, these results cannot be attributed to differences in RGC numbers, which were comparable between conditions, suggesting that the deficit likely arises from impaired synaptic connectivity or altered neuronal excitability. Notably, previous studies have hinted that retinal dysfunction can precede overt cell loss in splicing-factor RP models—for instance, network hyper- or hypo-activity has been observed in retinal degeneration models when photoreceptor input is perturbed [96,97]. In our *PRPF31*-ROs, the dampened RGC firing at 2 months likely reflects early circuit deficiencies that compromise synaptic communication, which are probably due to the impaired cell organization observed previously. This functional deficit could imply that the investigated *PRPF31* mutation in ROs impacts not only photoreceptor viability but also the overall retinal network function.

**Müller glia activation in *PRPF31*-ROs**

The scRNAseq analysis provided evidence that Müller glia is the first cell type to respond robustly to *PRPF31* haploinsufficiency in ROs. At day 85, a time point when photoreceptor numbers were comparable between genotypes, the *PRPF31*-ROs data exhibited a four-fold increase in the Müller glia/progenitor population, with a reduction in bipolar cells and RGCs. It should be noted that signaling pathways that guide the proliferation and differentiation of retinal progenitors also influence the ability of Müller glia to become progenitor cells, and therefore gene annotations at this early stage cannot distinguish between Müller glia and progenitor cells [98]. Nevertheless, this increase in Müller glia was accompanied by a profound alteration in their transcriptional state, with upregulation of acute gliosis and metabolic support markers including glial fibrillary acidic protein (*GFAP*), vimentin (*VIM*), and the glutamate transporter *SLC1A3*. These altered cell proportions could be due to a delay in retinal differentiation, but this appears unlikely, as early-stage ROs showed normal timing and progression of retinal specification in both control and *PRPF31*-. Instead, our findings indicate that *PRPF31* deficiency in ROs could lead to an early onset of degeneration rather than postponed maturation. Time-course analyses or transcriptomic trajectory inference could be applied in the future to clarify this issue. Nevertheless, pathway enrichment analysis showed a significant overrepresentation of pathways related to cellular response to chemical stress, oxidative stress response, and gliogenesis in the *PRPF31*-ROs Müller glia at this early stage. This might be due to an early protective, high-demand state that is known to become maladaptive if sustained [57,99,100,101]. In fact, this early gliotic response might represent an attempt to buffer retinal stress (consistent with Müller cells’ known initial compensatory reaction in stressed retinas [99,100]) but may predispose to chronic inflammation if sustained [101,102,103]. The early gliotic state was also validated at the protein level through immunofluorescence, where *PRPF31*-ROs at D85 displayed a significant increase in the percentage of STAT3+ cells compared to control ROs [104,105]. Indeed, JAK/STAT3 signaling was shown to be required to initiate Müller cell gliosis after retinal injury [106,107]. Similarly, the percentage of S100B+ cells was increased in *PRPF31*-ROs. S100B is a calcium-binding protein that it is shown to be upregulated by stressed Müller glia in the retina, driving neuroinflammation and potentially contributing to neuronal damage [108,109,110]. In diabetic retinopathy models, S100B levels become significantly elevated in Müller glia alongside GFAP during the initial gliotic response [63]. However, reactive Müller cells may enter a “detrimental” phase in which they secrete pro-inflammatory cytokines (e.g., TNF-α) and other cytotoxic factors, while losing homeostatic functions (e.g., glutamate uptake), thereby creating a hostile environment for neurons [111,112]. Indeed, blocking Müller/astrocyte intermediate filament polymerization or Stat3-dependent signaling attenuates glia-derived TNF-α and other stress signals, resulting in reduced retinal neuron apoptosis and overall pathology [103,113,114]. Thus, our scRNA-seq data on ROs indicate that Müller glial reactivity might initially be protective but, if sustained, could accelerate photoreceptor degeneration—a pattern consistent with reports from other retinal degeneration models [114,115].

**Inner retinal Dysfunction**

In parallel with Müller glial activation, we identified significant and early dysfunction in the inner retinal neuronal populations (RGCs and BCs). At day 85, scRNAseq data on *PRPF31*-ROs showed an upregulation of classical RGC markers, together with axonogenesis- and synapse-related genes [65,66,67,116]. This suggests that *PRPF31*-RO-derived RGCs activate developmental and plasticity programs in an attempt to consolidate neuronal identity and connectivity. However, this pattern may also reflect a stress-induced state: persistent GAP43 and SNAP25 activation has been linked to axonal instability and maladaptive remodeling under pathological conditions [117]. This transient “hyper-activation” is consistent with developmental asynchrony, in which RGCs attempt to compensate for the impaired splicing-related regulation of maturation pathways [13,20,77]. It should be noted that RGC pathology is typically considered a late consequence of photoreceptor degeneration [118,119], but our data on a patient RO model of RP suggests that this remodeling may commence surprisingly early in *PRPF31*-RP. Interestingly, the scRNAseq analysis showed a prolonged expression of RGC markers in our ROs, but we failed to detect specific RGC markers at the protein level or electrophysiological activities at late stage ROs. In fact, the absence of retrograde input from brain targets remains a limitation for RGC maturation and long-term maintenance in vitro [120,121]. The presence of RGC mRNA but not proteins at this late stage might be attributed to post-transcriptional modifications or low-level mRNA expression. Further analysis using spatial transcriptomics could clarify this discrepancy.

The interneuron data showed evidence of subtype-specific vulnerability and temporal mis-regulation in *PRPF31*-ROs. In controls, ON-bipolar cells data at D285 showed a coordinated induction of the canonical cascade, supporting mGluR6–TRPM1-mediated signaling [122,123,124]. *PRPF31*-ROs gene expression, however, failed to sustain this program and paradoxically upregulated *TRPM1* in the absence of *GRM6* and its partners. Such uncoupling, previously linked to TRPM1 mislocalization in degenerating retinas [55], likely renders ON signaling ineffective. OFF-bipolar cell expression showed an early skew at D85 but failed to establish the mature OFF identity program seen in controls at D285. This imbalance—early OFF bias coupled with loss of ON and OFF maturation—is consistent with previous studies showing that retinal degeneration destabilizes ON pathways while transiently maintaining OFF responses [55,125,126]. Such dynamics likely contribute to the weakened excitatory drive to RGCs, and the functional deficits observed in our *PRPF31*-ROs. These findings collectively indicate that inner retinal network dysfunction might act as a primary pathogenic event in our RP11 in vitro model.

**Photoreceptor Degenerative Expression Dynamics in *PRPF31*-ROs**

The ultimate and defining feature of RP11 is the progressive degeneration of rod and cone photoreceptors [2,7,127]. The scRNA-seq data on day 285 showed a severe, five-fold decrease in cone markers and a significant reduction in the remaining rod population in *PRPF31*-ROs. This cellular attrition preceded a near-complete downregulation of the entire phototransduction cascade, as well as key maintenance transcription factors, suggesting a breakdown of cell fate maintenance under degenerative distress [128,129,130]. Pathway enrichment analysis summarized this functional collapse in ROs. By day 285, control photoreceptors data were highly enriched for pathways related to the detection of visible light, whereas in *PRPF31*-ROs, these were replaced by pathways for cell death in response to oxidative stress and inflammatory response. Interestingly, at D85, *PRPF31*-ROs cones exhibited a transient, paradoxical upregulation of developmental regulators like RXRγ and TRβ2, likely representing an initial, stressed attempt to drive differentiation that ultimately failed [130,131]. Inflammatory pathways remained active as well, reinforcing the notion that a chronic inflammatory milieu might be developed in our *PRPF31*-RO model. Notably, inflammatory mediators such as IL-6 and TNF-α have been found to be elevated in *PRPF31*-deficient ROs under stress [8,132], and pro-inflammatory pathways are activated in other retinal degeneration models (including rodent RP), where they hasten photoreceptor demise [133].

Taken all together, our data suggests, at least in our RO model, that inner retinal cells are the primary targets of *PRPF31*-mediated distress, actively contributing to the pathogenic microenvironment long before the outer retina collapses. These findings portray a sequence in which an initial, intrinsic cellular distress (due to an ubiquitous splicing-factor mutation) is met by compensatory glial and neuronal responses, but over time this balance tips into a state of chronic oxidative stress, inflammation, and structural failure that specifically devastates the photoreceptors. The inflammatory component in our RO model aligns with reports that anti-inflammatory agents can slow retinal degeneration [134]. Finally, our scRNAseq data on ROs revealing early Müller glial gene expression increase suggests that modulating glial reactions might influence disease outcome, given that Müller cells’ supportive functions can later turn detrimental [115].

**Limitations of the study**

Despite the valuable insights gained, this study has limitations that warrant consideration. Although our control data were comparable to human retina atlas and published ROs single-cell datasets, the absence of a comparison with an isogenic control line prevents definitive attribution of all observed phenotypic and transcriptomic differences solely to the *PRPF31* mutation. Also, the focus on a single pathogenic variant (c.1115_1125del11) limits the generalizability of our findings across the genetic heterogeneity and the large range of phenotypic variability of *PRPF31*-associated RP in patients [135]. Moreover, although our organoid model captures key features of retinal development and degeneration, it lacks in vivo validation and certain in vivo components such as vascular supply, potentially omitting important aspects of the disease microenvironment. Functional assessments were primarily limited to early electrophysiology, and long-term evaluations of photoreceptor activity or therapeutic rescue were not performed. To further strengthen the model, future work should include gene-edited isogenic controls, examine additional *PRPF31* variants, and increase organoid complexity through microglia integration or assembloid approaches. Functional rescue or knockdown experiments will also be crucial to directly confirm that the observed phenotypes result from *PRPF31* deficiency. Additional functional assays—such as light-response testing, calcium imaging, and metabolic profiling—will be critical to evaluate photoreceptor integrity.

## 5. Conclusions

In conclusion, our results support a specific model of RP11 in which a primary molecular defect (*PRPF31* c.1115_1125 del11 haploinsufficiency) precipitates the degeneration of the most vulnerable retinal cells—the rod and cone photoreceptors—while simultaneously eliciting compensatory or modulatory responses in other retinal cell types. In our RO model, Müller glia/progenitors, RGCs, and bipolar cells engage in an interactive distress that may initially buffer the retina but could eventually contribute to chronic inflammation and neurodegeneration, thereby influencing the pace of disease progression. Future investigation involving additional *PRPF31* iPSC lines and their corresponding isogenic controls, together with more complex in vitro models such as microglia integration, assembloids, or microfluidics models, could help to further elucidate the role of *PRPF31* in inflammation and retinal degeneration.

## Data Availability

The datasets used and/or analyzed during the current study are available from the corresponding author on reasonable request. The dataset we generated for these studies is available through the NCBI GEO accession GSE307923 (https://www.ncbi.nlm.nih.gov/geo/query/acc.cgi?acc=GSE307923 (accessed on 17 June 2025)).

## Supplementary Materials

The following supporting information can be downloaded at https://www.mdpi.com/article/10.3390/biomedicines14010045/s1, Figure S1: Characterization of PRPF3 and control hiPSCs; Figure S2: Flow cytometry of *PRPF31* and control hiPSCs and PCR Amplification of *PRPF31* gene; Figure S3: Reduced *PRPF31* protein expression in *PRPF31*-ROs; Figure S4:—Cleaved Caspase-3 localization reveals increased inner retinal apoptosis without necrotic core formation in *PRPF31*-ROs; Figure S5: Raster plots of spontaneous spiking activity; Figure S6: Single-cell transcriptomic UMAP visualization of ROs; Figure S7: Annotation of retinal cell populations in control ROs against a reference dataset; Figure S8: Global heatmap of retinal cell-type marker expression. Table S1: List of primary antibodies; Table S2: List of secondary antibodies; Table S3: List of primer sequences; Table S4: Cell type—specific markers used for scRNAseq annotation.

## Abbreviations

The following abbreviations are used in this manuscript:

adRP	Autosomal Dominant Retinitis Pigmentosa
arRP	Autosomal Recessive Retinitis Pigmentosa
BC	Bipolar Cell
DAPI	4′,6-diamidino-2-phenylindole
EB	Embryoid Bodies
GFR	Growth Factor Reduced
GCL	Ganglion Cell Layer
GO	Gene Ontology
hiPSC	Human Induced Pluripotent Stem Cell
IF	Immunofluorescence
KOSR	Knock-Out Serum Replacement
MEA	Micro Electrode Array
NGS	Next Generation Sequencing
NIM	Neural Induction Media
NR	Neural Retina
OLM	Outer Limiting Membrane
ONL	Outer Nuclear Layer
OPV	Organic Photo-Voltaic
PBS	Phosphate-Buffered Saline
PFA	Paraformaldehyde
PR	Photoreceptor
RC1	Retinal Constituent 1
RC2	Retinal Constituent 2
RDM	Retinal Differentiation Media
RGC	Retinal Ganglion Cell
RO	Retinal Organoid
RP	Retinitis Pigmentosa
RPE	Retinal Pigment Epithelium
scRNAseq	Single-cell RNA Sequencing
SEM	Standard Error of the Mean
ssGSEA	single-sample Gene Set Enrichment Analysis
TBS	TRIS-Buffered Saline
UMAP	Uniform Manifold Approximation and Projection
UPR	Unfolded Protein Response
WT	Wild Type
xlRP	X-linked Retinitis Pigmentosa
