# Supplementary material for: Single-Cell Transcriptomics on PRPF31-Mutated Retinal Organoids Reveal Early Müller Glial Activation and Progressive Photoreceptor Degeneration
Source: Biomedicines. 2025 Dec 24;14(1):45. doi: 10.3390/biomedicines14010045 (PMC12837672; doi:10.3390/biomedicines14010045)
Supplement: Supplementary file 1 [file biomedicines-14-00045-s001.zip › biomedicines-4021598-supplementary.pdf]

Supplementary Data

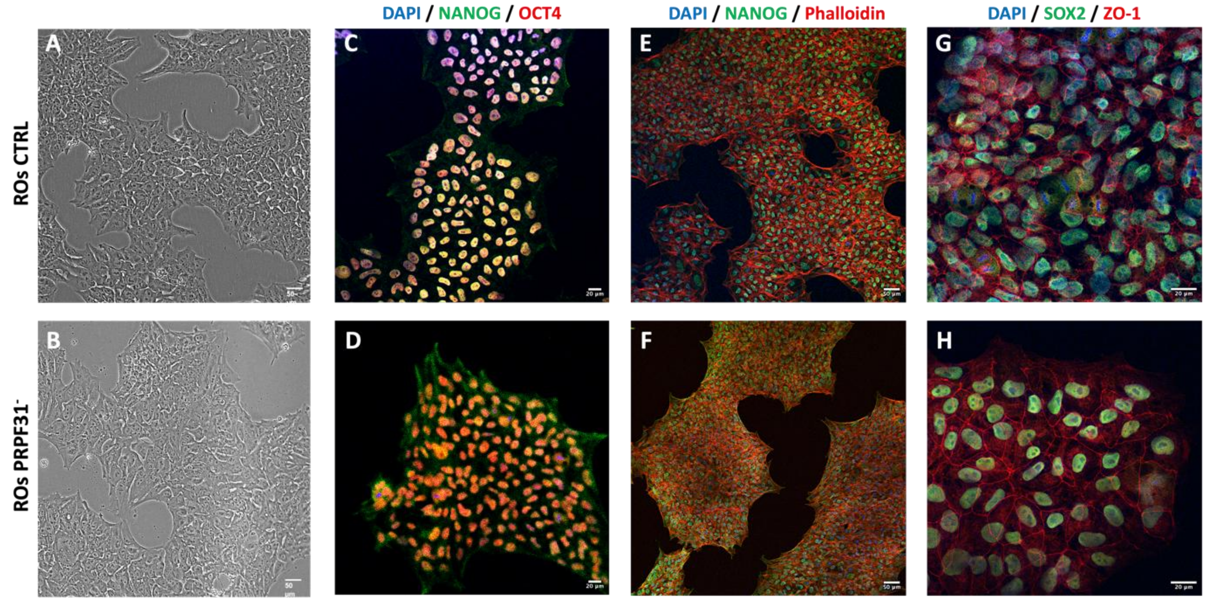

Figure S1 - Characterization of PRPF3 and control hiPSCs: (A–B) Phase-contrast images showing similar undifferentiated morphology of control (A) and PRPF31-mutant (B) hiPSC colonies. (C–D) Immunofluorescence staining for pluripotency markers NANOG (green) and OCT4 (red) with DAPI nuclear counterstain (blue) confirms robust co-expression of core stemness factors in both control (C) and PRPF31-mutant (D) hiPSC lines. (E–F) Staining with NANOG (green) and Phalloidin (red; F-actin marker) reveals intact cytoskeletal organization in control (E) and mutant (F) colonies. (G–H) Expression of SOX2 (green) and ZO-1 (red) further supports maintenance of pluripotency and epithelial colony morphology in both control (G) and mutant (H) hiPSCs. Scale bar: 50 μm (A–B, E–F), 20 μm (C–D, G–H).

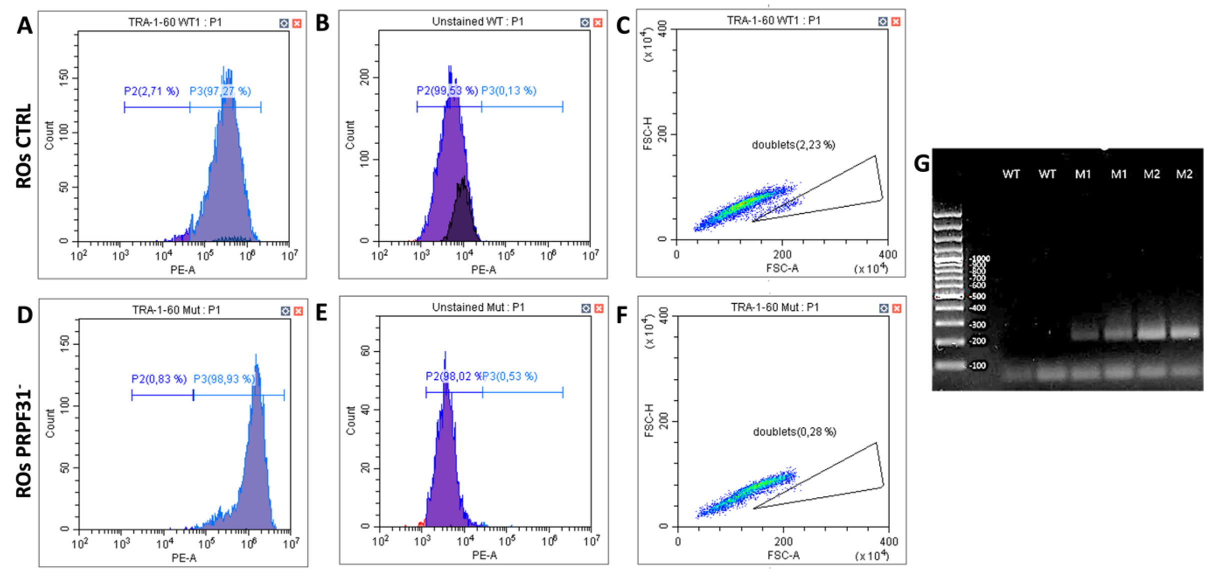

Figure S2 - Flow cytometry of PRPF31 and control hiPSCs and PCR Amplification of PRPF31 Gene: (A-F) Flow cytometry analysis of TRA-1-60 surface marker expression confirms high pluripotency marker expression in both

control (A-C) and PRPF31 (D-F) hiPSC lines. Histogram showing ~97% TRA-1-60-positive cells in control (A); ~99% TRA-1-60-positive cells in PRPF31- line (D). (B; E) Unstained controls confirm specificity of TRA-1-60 staining. (C; F) Scatter plots show low percentage of doublets, ensuring high sample purity. (G) PCR-based amplification analysis reveals expected PRPF31 mutation in the PRPF31 hiPSCs (M1, M2) compared to control (WT) hiPSCs, confirming integrity of the patient-specific cell lines.

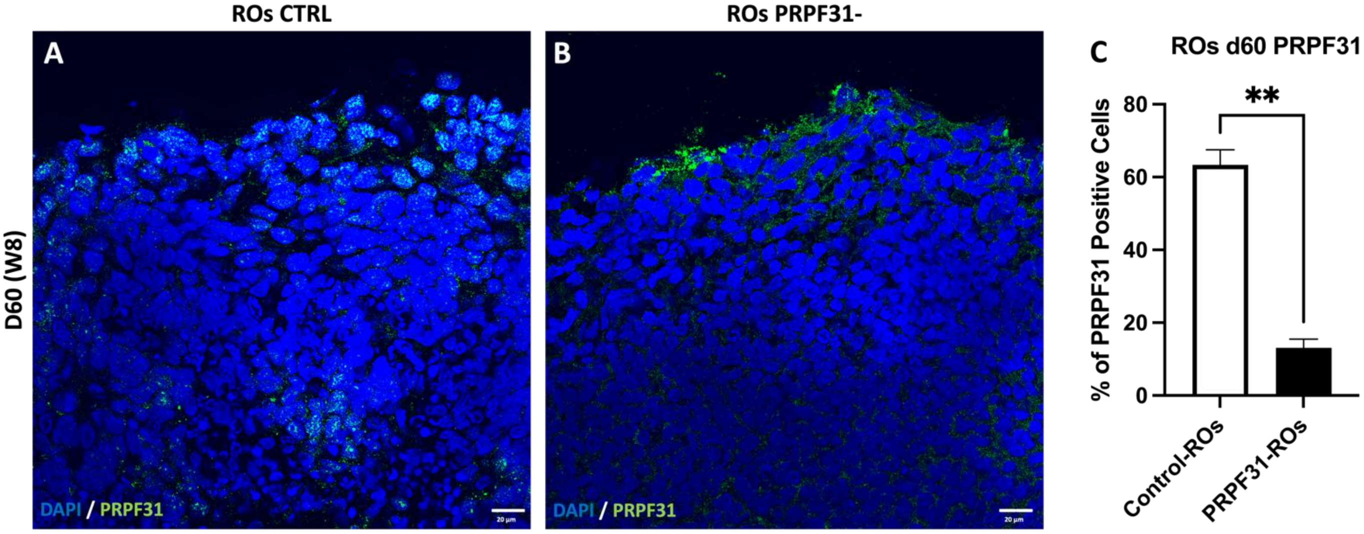

Figure S3 – Reduced PRPF31 protein expression in PRPF31-ROs: Immunofluorescence analysis of ROs showing PRPF31 protein expression (green) with DAPI nuclear counterstaining (blue). (A) Control ROs at day 60 (D60) showing nuclear PRPF31 localization. (B) PRPF31⁻ ROs immunofluorescence at D60 show a reduction in PRPF31 signal compared to controls. (C) Quantification of PRPF31-positive nuclei at D60 reveals a significant decrease in PRPF31 protein expression in PRPF31⁻ ROs relative to control ROs (**$p < 0.01$; n = 3–5 organoids per condition). Scale bar = 20 µm

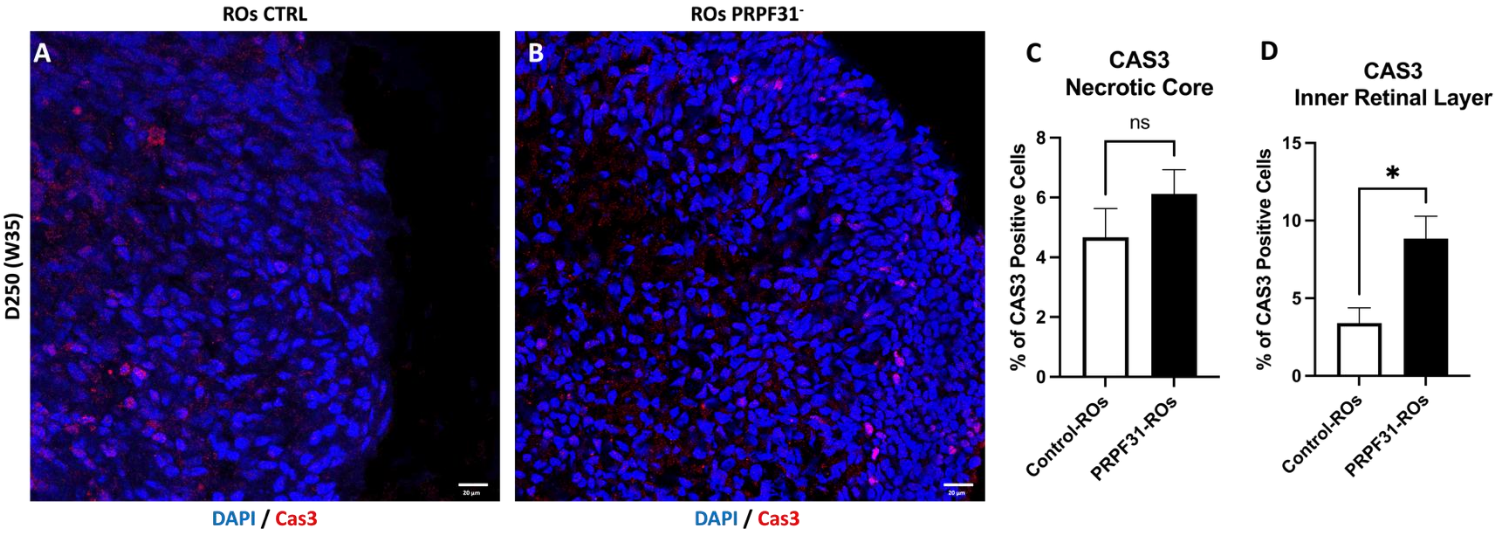

Figure S4 – Immunofluorescence staining for cleaved Caspase-3 (CAS3, red) and DAPI nuclear counterstaining (blue) in ROs at day 250 (D250). (A) Control ROs show sparse CAS3⁺ cells. (B) PRPF31⁻ ROs staining displays increased CAS3 immunoreactivity, predominantly localized to the inner retinal layer (IRL). (C) Quantification of CAS3⁺ cells within the central necrotic core region shows no significant difference between control and PRPF31⁻ ROs ($p = 0.3095$; n = 3–5 organoids per condition). (D) Quantification of CAS3⁺ cells within the inner retinal layer

reveals a significant increase in $PRPF31^{-}$ ROs compared to controls (*$p < 0.05$; n = 3–5 organoids per condition). These data indicate that elevated CAS3 signal in $PRPF31^{-}$ ROs reflects localized inner retinal apoptosis rather than widespread cell death associated with necrotic core formation. Scale bar = 20 µm.

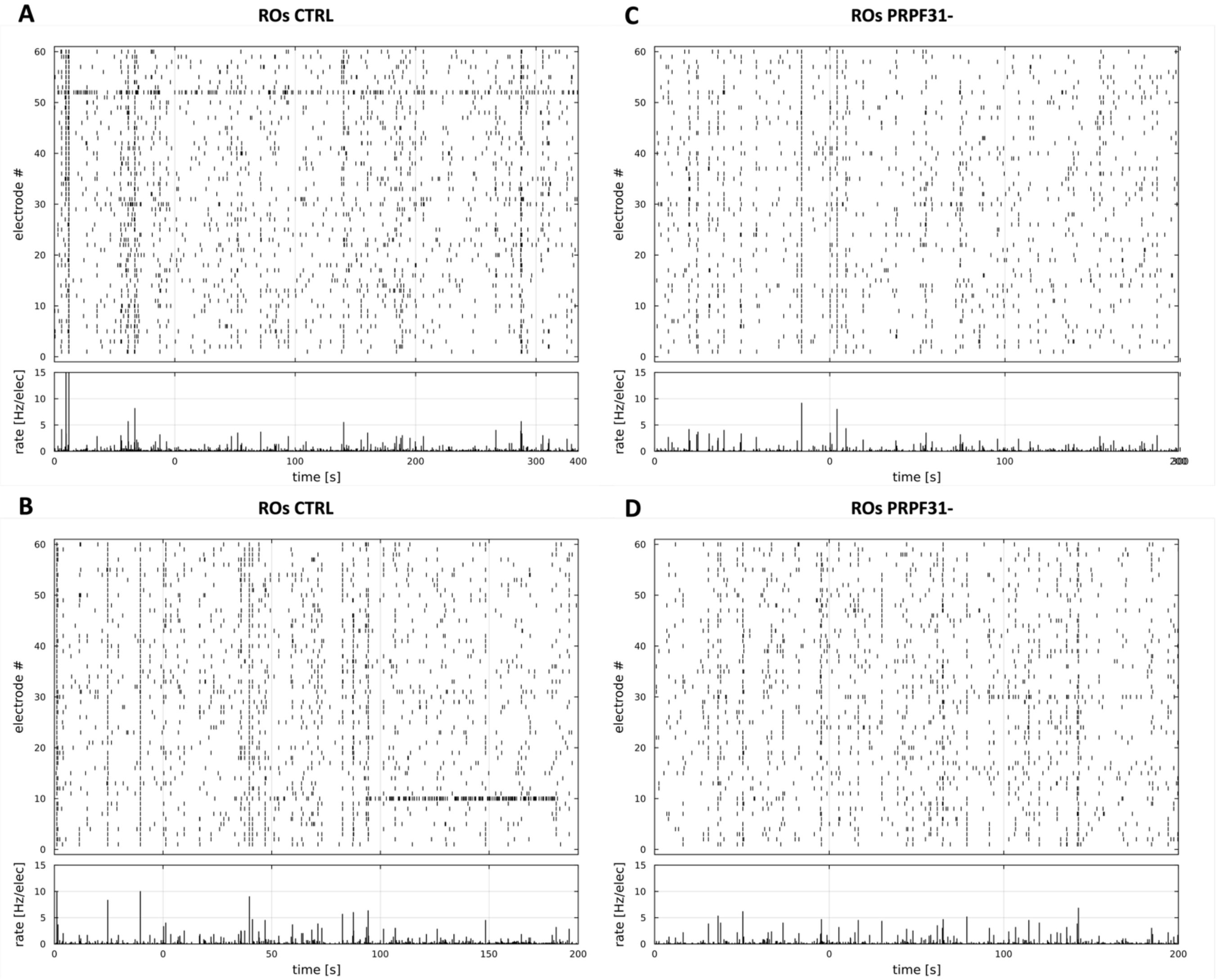

Figure S5 – Raster plots of spontaneous spiking activity: Network level analysis of MEA electrodes in control (A, B) and PRPF31-ROs (C, D)**.** Each dot represents a spike event at a specific electrode and time point. The accompanying histo-grams display the overall firing rate across electrodes. Control organoids exhibit robust, wide-spread spiking across multiple channels, whereas PRPF31-ROs show reduced spike density and lower overall firing rates, indicating impaired spontaneous neuronal network activity.

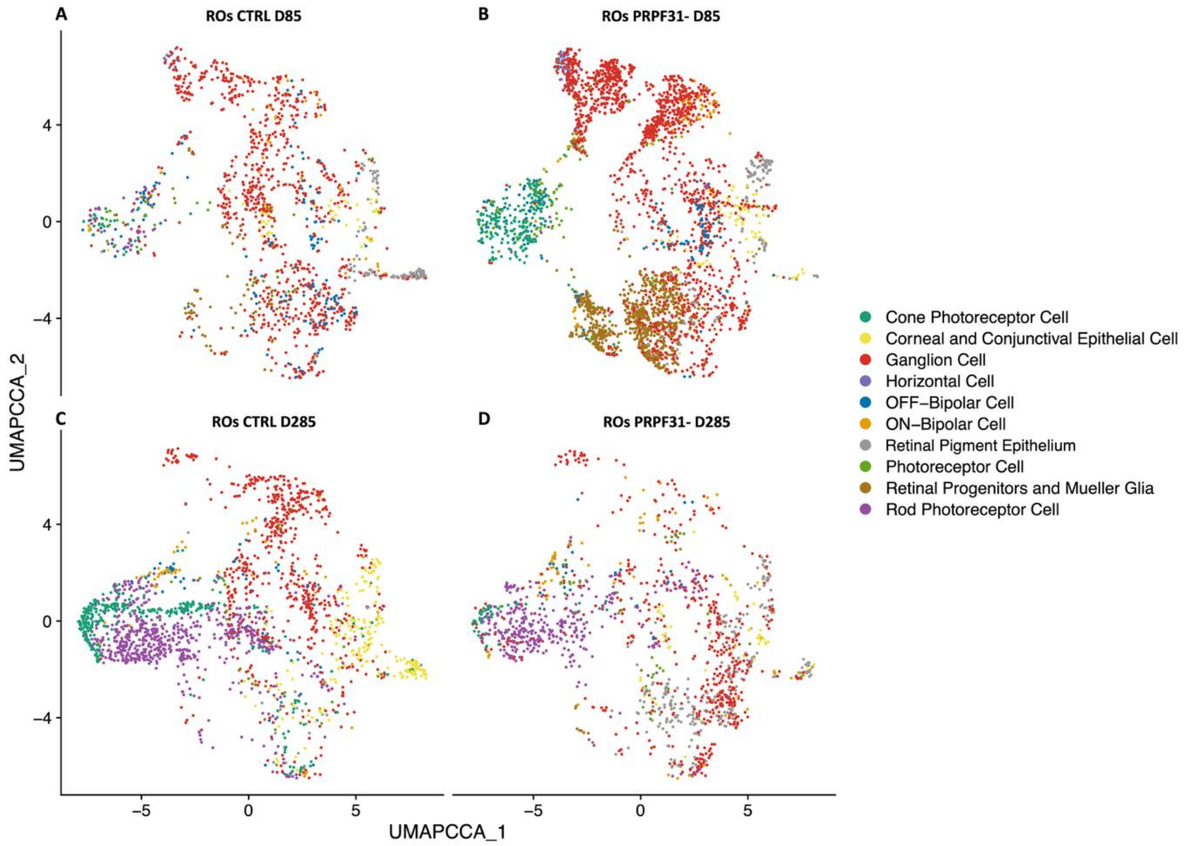

Figure S6 – Single-cell transcriptomic UMAP visualization of ROs: UMAP plots showing clustering of all major retinal cell populations in control and PRPF31-ROs at **day 85 (D85)** and **day 285 (D285)**. (A,B) At D85, both control(A) and PRPF31- ROs (B) contain all major retinal lineages; however, PRPF31-ROs display a pronounced expansion of Müller glia/progenitor clusters, reduced ganglion cell fraction, relative cone enrichment, and near absence of rods. (C,D) At D285, control ROs (C) show robust and balanced photoreceptor clusters (rods and cones), while PRPF31- ROs (D) exhibit severe cone loss, reduced rod populations, and a relative increase in ganglion and retinal pigment epithelium clusters, indicating disease-associated remodeling.

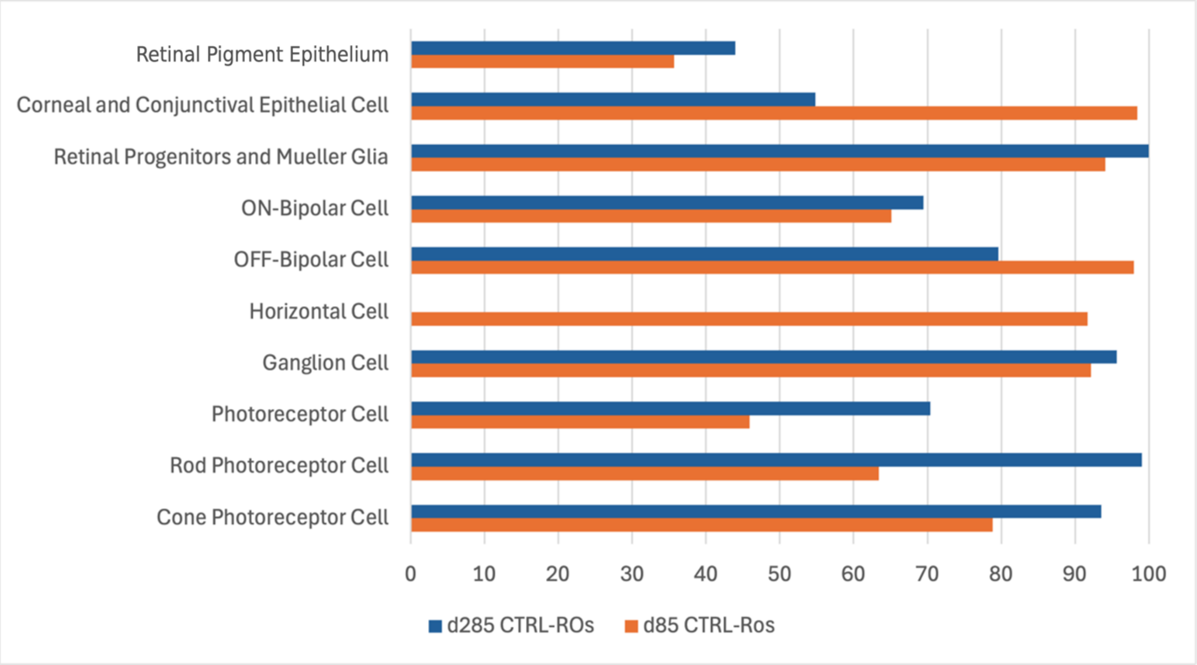

Figure S7 – Annotation of retinal cell populations in control ROs against a reference dataset: Bar graph showing the distribution of annotated retinal cell populations from control organoids at day 85 (orange) and day 285 (blue) mapped onto a reference single-cell retinal dataset (Jones et al., 2023 - GSE220624). The reference dataset included Interneurons, Photoreceptors (PR), Progenitors and RPE. Control-ROs populations have been compared as follows: Cone PR, Rod PR, PR vs. PR; RGCs, Horizontal cells, OFF-BCs, ON-BCs vs Interneurons; Müller Glia vs Progenitors; Corneal/Conjunctival, Retinal Pigment Epithelium vs RPEs.

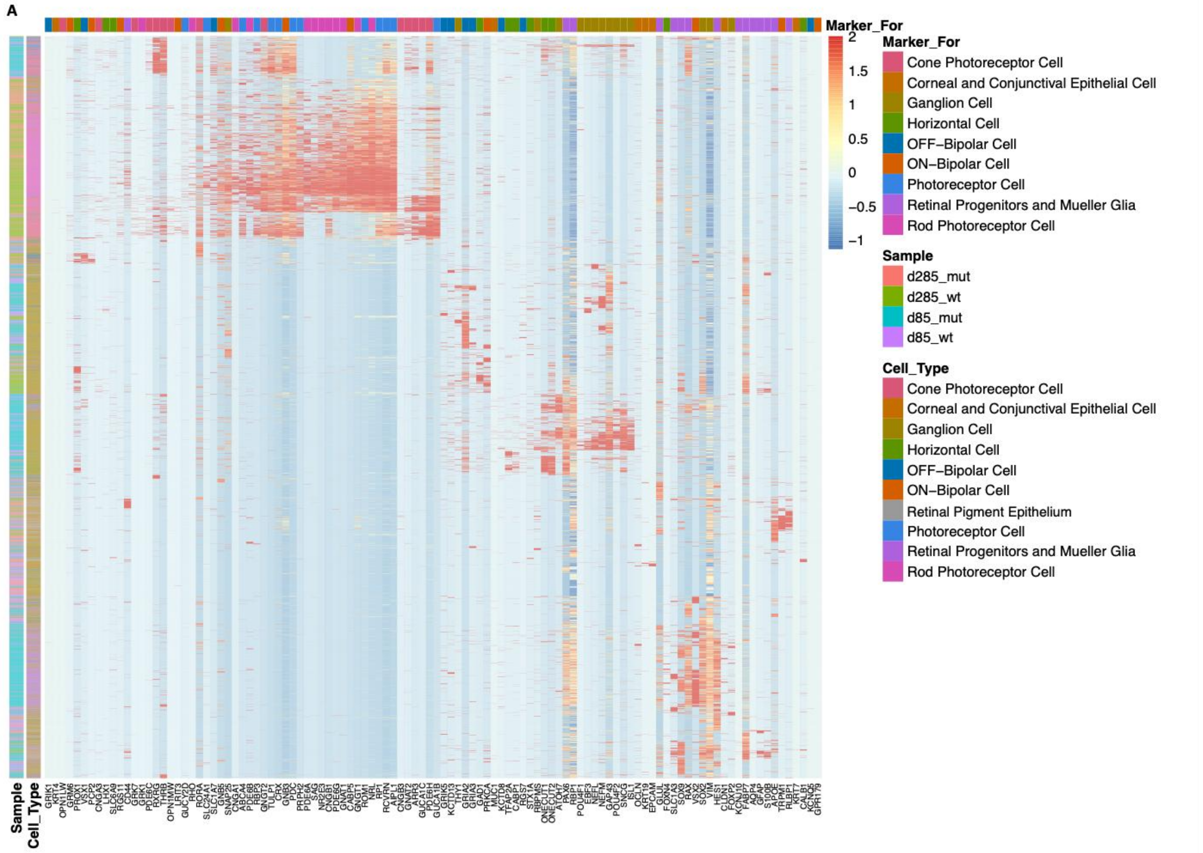

Figure S8 – Global heatmap of retinal cell-type marker expression: Expression profiles of canonical marker genes across all organoids (d85 and d285, WT and PRPF31-mutant). Columns represent cells and rows represent marker genes, color-coded by scaled expression (blue = low, red = high). Distinct transcriptional signatures identify all major retinal cell types, with condition- and stage-dependent shifts in photoreceptor and Müller glial markers.

Table S1. List of Primary Antibodies

Antibody	Species	Concentration	Manufacturer	Catalog #	RRID
Oct-4A	Rabbit	1:400	Cell Signaling Technology	2840	AB_2167691
ZO-1	Rabbit	1:200	Proteintech	21773-1-AP	AB_10733242
Nanog	Mouse	1:200	Santa Cruz Biotechnology	sc-293121	AB_2665475
Sox-2	Mouse	1:200	Santa Cruz Biotechnology	sc-365823	AB_10842165
PAX6	Rabbit	1:100	Proteintech	12323-1-AP	AB_2159695
TUJ1 (TUBB3)	Mouse	1:500	Proteintech	66375-1-Ig	AB_2814998
OTX2	Mouse	1:400	Santa Cruz Biotechnology	sc-514195	AB_2921699
VSX2	Rabbit	1:400	Proteintech	25825-1-AP	AB_2880257
POU4F2	Rabbit	1:200	Proteintech	55042-1-AP	AB_10888633
Recoverin	Rabbit	1:400	Proteintech	10073-1-AP	AB_2178005
CRX	Mouse	1:400	Santa Cruz Biotechnology	sc-377138	-
Arrestin C	Rabbit	1:400	Proteintech	11100-2-AP	AB_2289959
L/M Opsin	Rabbit	1:200	Kerafast	EDK101	-
Rhodopsin	Mouse	1:200	Absolute Antibody	Ab00337-10.7	-
Phalloidin 555	Conjugated	1:200	Invitrogen	A30106	-
S100B	Rabbit	1:400	Proteintech	15146-1-AP	AB_2254244
STAT3	Rabbit	1:400	Proteintech	10253-2-AP	AB_2302876
PRPF31	Rabbit	1:200	Proteintech	27750-1-AP	AB_2918130
Caspase 3	Rabbit	1:400	Proteintech	25128-1-AP	AB_3073913

Table S2. List of Secondary Antibodies

Antibody	Concentration	Manufacturer	Catalog #	RRID
Alexa Fluor 488 Goat anti-rabbit	1:1000	Invitrogen	A-11034	AB_2576217
Alexa Fluor™ 488 Goat anti-Mouse	1:1000	Invitrogen	A-11029	AB_2534088
Alexa Fluor™ 546 Donkey anti-Rabbit	1:1000	Invitrogen	A-10040	AB_2534016
Alexa Fluor™ 555 Goat anti-Mouse	1:1000	Invitrogen	A-21424	AB_141780
Alexa Fluor™ 647 Goat anti-Mouse	1:1000	Invitrogen	A-21235	AB_2535804
Alexa Fluor™ 647 Donkey anti-Goat	1:1000	Invitrogen	A32849	AB_2762840
CoraLite® Plus 750-Goat Anti-Mouse	1:1000	Proteintech	RGAM006	AB_3073504

Table S3. List of Primer sequences

Gene	Forward (F) / Reverse (R)	Sequence
GAPDH	F	CTGGTAAAGTGGATATTGTTGCCAT
GAPGH	R	TGGAATCATATTGGAACATGTAAACC
OPN1MW	F	GAACCAGGTCTATGGCTACTTCG
OPN1MW	R	TCTCACATTGCCAAAGGGCTT
CRX	F	TCCAGGGTTCAGGTTTGGTT
CRX	R	CATCTGTGGAGGGTCTTGGG
RECOVERIN	F	TCTACGACGTGGACGGTAACG
RECOVERIN	R	CGTCCTCGGGAGTGATCATT
RHODOPSIN	F	GGGAGAACCATGCCATCAT
RHODOPSIN	R	TCGTCTCCGTCTTGGA
ARRESTIN 3	F	CCCAGAGCTTTGCAGTAACC
ARRESTIN 3	R	CACAGGACACCATCAGGTTG

Table S4. Cell type – specific markers used for scRNAseq Annotation

Retinal Cell Population	Gene Markers
Photoreceptor Cell	CRX, RCVRN, RBP3, PRPH2, ROM1, ABCA4, GUCA1B, PDC, GUCY2D, TULP1, RP1, AIPL1, SLC24A1
Rod Photoreceptor Cell	RHO, GNAT1, SAG, PDE6A, PDE6B, CNGA1, CNGB1, NR2E3, NRL, ROM1, RCVRN, PDE6G, GRK1, GNGT1, ESRRB
Cone Photoreceptor Cell	ARR3, GNAT2, OPN1SW, OPN1MW, OPN1LW, PDE6C, PDE6H, CNGA3, CNGB3, GUCA1C, GUCA1B, GNGT2, RXRG, THRB, RORA, GRK7
Horizontal Cell	PROX1, LHX1, ONECUT1, ONECUT2, GJA10, CALB1, FOXN4, SLC6A9, TFAP2A, STX1A, CABP1

Ganglion Cell	RBPMS, SNCG, POU4F2, POU4F1, THY1, NEFL, NEFM, GAP43, ISL1, EBF3, ATOH7, OPN4, SNAP25, FOXP2, SOX2, VIM
ON-Bipolar Cell	GRM6, TRPM1, GPR179, LRIT3, NYX, PCP2, PRKCA, CABP5, RGS11, GNB3, VSX2, GNB5
OFF-Bipolar Cell	GRIK1, GRIK5, RGS7, KCNQ5, KCTD13, KCTD8, SLC1A7, VSX1, GRIA2, GRIA3
Mueller Glia / Progenitors	RLBP1, GLUL, RBP1, SLC1A3, KCNJ10, AQP4, VIM, SOX9, SOX2, HES1, VSX2, RAX, PAX6, FABP7, GFAP, S100B, CD44, APOE
Corneal / Conjunctival Epithelial Cell	KRT3, KRT12, KRT13, KRT19, KRT4, KRT7, KRT5, KRT14, KRT15, EPCAM, TACSTD2, PAX6, ALDH3A1, CLDN1, OCLN, MUC1, MUC4, MUC16
Retinal Pigment Epithelium	RPE65, BEST1, MITF, TYR, TYRP1, PMEL, DCT, LRAT, MERTK, CLDN19, TJP1
